# How effective are River Basin Management Plans in reaching the nutrient load reduction targets?

**DOI:** 10.1007/s13280-020-01393-x

**Published:** 2020-09-27

**Authors:** Mikołaj Piniewski, Sirkka Tattari, Jari Koskiaho, Olle Olsson, Faruk Djodjic, Marek Giełczewski, Paweł Marcinkowski, Marta Księżniak, Tomasz Okruszko

**Affiliations:** 1 Department of Hydrology, Meteorology and Water Management, Warsaw University of Life Sciences, Nowoursynowska 166, 02-787 Warsaw, Poland; 2 Finnish Environment Institute (SYKE), Latokartanonkaari 11, 00790 Helsinki, Finland; 3 Stockholm Environment Institute (SEI), Linnégatan 87D, 10451 Stockholm, Sweden; 4 Swedish University of Agricultural Sciences, Lennart Hjelms väg 9, 75007 Uppsala, Sweden; 5 Department of Remote Sensing and Environmental Assessment, Warsaw University of Life Sciences, Nowoursynowska 166, 02-787 Warsaw, Poland

**Keywords:** Baltic Sea, Best Management Practices, Nutrient load, River Basin Management Plans, SWAT model, Targeting of measures

## Abstract

Riverine nutrient loads are among the major causes of eutrophication of the Baltic Sea. This study applied the Soil & Water Assessment Tool (SWAT) in three catchments flowing to the Baltic Sea, namely Vantaanjoki (Finland), Fyrisån (Sweden), and Słupia (Poland), to simulate the effectiveness of nutrient control measures included in the EU’s Water Framework Directive River Basin Management Plans (RBMPs). Moreover, we identified similar, coastal, middle-sized catchments to which conclusions from this study could be applicable. The first modelling scenario based on extrapolation of the existing trends affected the modelled nutrient loads by less than 5%. In the second scenario, measures included in RBMPs showed variable effectiveness, ranging from negligible for Słupia to 28% total P load reduction in Vantaanjoki. Adding spatially targeted measures to RBMPs (third scenario) would considerably improve their effectiveness in all three catchments for both total N and P, suggesting a need to adopt targeting more widely in the Baltic Sea countries.

**Electronic supplementary material:**

The online version of this article (10.1007/s13280-020-01393-x) contains supplementary material, which is available to authorized users.

## Introduction

Aquatic eutrophication caused by excessive loads of nutrients transported by rivers to the Baltic Sea remains the primary environmental issue despite investments aimed at the reduction of pollution from both point and diffuse sources within its drainage basin. The Baltic Sea is particularly vulnerable to waterborne nutrient loads as a result of the following factors: (1) large size of the basin compared to the sea area; (2) long freshwater renewal time; and (3) limited water exchange with the North Sea.

In 1974, the Helsinki Convention established an organisation called the Helsinki Commission (HELCOM). All the Baltic Sea coastal states are parties to HELCOM and the Helsinki Convention, together with the European Union (EU). A program for regular data collection has been set up within HELCOM, and official hot-spot “problem areas” have been identified. Until the 1980′s, the share of point sources, primarily from wastewater treatment plants, was high in many countries. Therefore, the focus was on reducing such sources. While point sources have been successfully addressed, diffused sources of nutrient pollution related to e.g. large-scale animal farm production and other agricultural activities and are yet to be resolved.

In 2007, the Baltic Sea Action Plan (BSAP) was adopted by HELCOM’s contracting parties (i.e. countries and the European Union). BSAP is an ambitious program aimed at restoring good ecological status of the Baltic marine environment by 2021. It relies on the latest scientific knowledge and innovative management approaches to strategic policy throughout the Baltic Sea region (BSR). The BSAP is not static—it is regularly updated in annual ministerial meetings.

HELCOM member states are also obliged to implement the EU’s Water Framework Directive’s (WFD) regional River Basin Management Plans (RBMPs). The 4th planning period, ending in 2021, is currently underway. The objective of RBMPs to achieve good status of surface waters is similar to that of BSAP, but in addition to coastal areas it covers inland waters and groundwaters. Moreover, RBMPs define measures needed to achieve this target. Meeting this objective, as well as that of BSAP, however, has proven difficult, and not all water bodies and coastal waters of BSR will reach good status for the period of 2021–2027 (e.g. Schumacher 2012; Knuuttila et al. 2017; Bohman 2018).

HELCOM has set specific targets for reducing nutrient loads. In 2007, original preliminary reductions of phosphorus (P) and nitrogen (N) were stipulated, compared to the input in the reference period of 1997–2003 (HELCOM 2007). In 2013, the figures were revised based on a new and more complete dataset. The new Maximum Allowable Inputs (MAIs) were set at different levels, and Country Allocated Reduction Targets (CARTs) were established for different sea areas and countries of the Baltic Sea (HELCOM 2013; Table 1).

**Table 1:** Country-specific N and P mean annual loads as well as CART and RBMP reduction targets

Country		PLC (’06; ’14) annual load^a^	CART^b^ target reduction	RBMP^c^ target reduction	CART reduction
		[t year^−1^]	[t year^−1^]	[t year^−1^]	[%]
Finland	N	92 771	3030	6600	3.3
	P	3585	356	440	10
Poland	N	188 499	43 610	na	23.1
	P	12 214	7480	na	61
Sweden	N	120 323	9240	13 720	7.7
	P	3463	530	1167	16

^a^Pollution Load Compilation, estimation based on PLC reports regarding years 2006 and 2014

^b^Country Allocated Reduction Targets

^c^River Basin Management Plans

Over the recent decades, hydrological and water quality modelling has become an important tool to improve the understanding of the effects of various types of pollution sources on nutrient loads at different spatial scales. This is particularly apparent in BSR due to many past and ongoing projects aimed at quantifying the effectiveness of both already implemented and suggested measures. Models are used for estimating nutrient loads (Hesse et al. 2015; Huttunen et al. 2015; Thodsen et al. 2017; Olesen et al. 2019) and identifying hot spots with high nutrient losses (Thodsen et al. 2015; Andersen et al. 2016; Djodjic and Markensten 2019). They are also used for assessing the effectiveness of measures such as buffer zones (Piniewski et al. 2015), constructed wetlands (Arheimer and Pers 2017), and catch crops (Konrad et al. 2014). In the conditions of intensifying climate change, models are also increasingly frequently used for the assessment of its interactions with land use, management practices, and effects on water quality. It should be emphasised that this type of research usually focuses on longer time horizons reaching 2050 or even 2100 (Piniewski et al. 2014; Huttunen et al. 2015; Olesen et al. 2019). In addition to climate change, socio-economic aspects have been considered with more attention in recent studies predicting trends in catchment management and assessing nutrient loads (Pastuszak et al. 2014; Huttunen et al. 2015; Olesen et al. 2019).

The objectives of this paper are as follows: (1) Estimation of factors affecting nutrient loads for the purpose of modelling their changes in a 15-year perspective in three medium-sized catchments flowing into the Baltic Sea; (2) Evaluation of the effectiveness of catchment-specific nutrient load control measures included in the existing RBMPs in a 15-year perspective; (3) Evaluation of how spatial targeting of measures could enhance the effectiveness of RBMPs in three case study catchments.

## Materials and methods

### Case study catchments

Three medium-sized coastal catchments situated in Finland, Sweden and Poland were selected for focused modelling studies and comparisons.

**Fig. 1:**
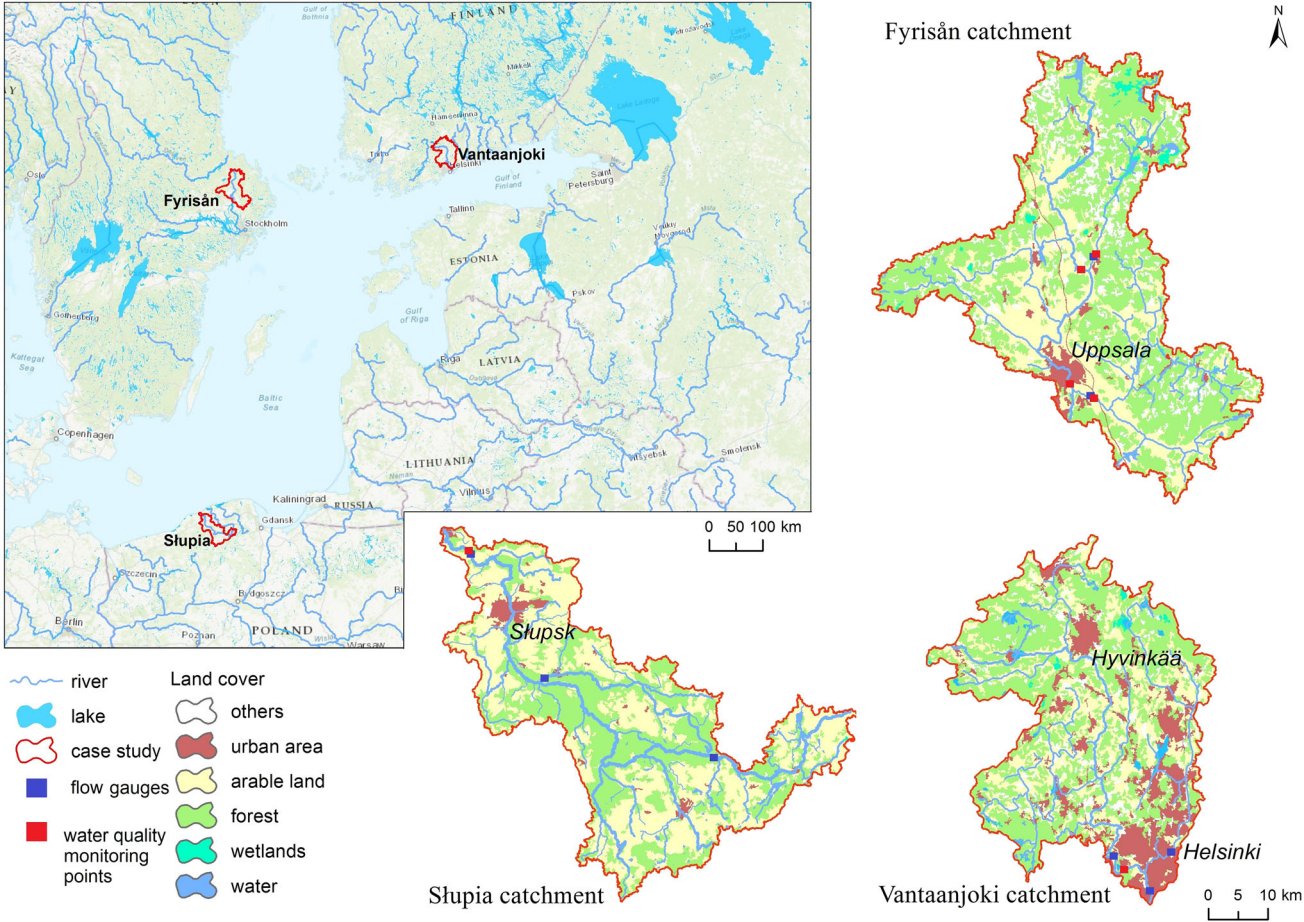
Location of case study catchments

The Vantaanjoki (101 km) in southern Finland drains a 1688 km^2^ catchment, and flows into the Gulf of Finland (Fig. 1). The climate is characteristic of the boreal zone and belongs to the *Dfb* (warm-summer humid continental) class according to the Köppen-Geiger system (Beck et al. 2018). Mean precipitation is 660 mm year^−1^ and mean water flow is 11.5 m^3^ s^−1^, ranging between 0.7 and 125 m^3^ s^−1^. The elevation ranges between 0 and 150 m a.s.l. The highest parts and reliefs of the catchment occur in the north and are mostly covered by forest. Agricultural land and clay soils are predominant in the middle and southern part of the catchment which is flat or slightly hilly. Land use includes forests (53%), agriculture (25%), and urban areas (18%), and water covers 2% of its area. The city of Helsinki with approximately 655 000 inhabitants is located in the south-eastern corner of the catchment.

The Fyrisån catchment (1982 km^2^) is located in the south-eastern part of Sweden. The approximately 80 km long Fyrisån is part of the wider Norrström drainage basin, and discharges into Lake Mälaren which has its outlet through Stockholm into the Baltic Sea. The climate and vegetation are characteristic of the boreal zone like in the Vantaanjoki catchment, with climate also classified as *Dfb*. Mean annual precipitation is approximately 550–600 mm year^−1^. Land use in the catchment is distributed between forests (60%), agriculture (32%), wetlands (4%), lakes (2%), and urban areas (2%). Soils in the catchment are primarily glacial tills on forest land, whereas clay soils dominate in agricultural regions. Altitudes range from 1 to 117 m a.s.l, and mean water flow in the Fyrisån is approximately 13 m^3^ s^−1^, ranging between 1 and 140 m^3^ s^−1^. The urban area is dominated by the city of Uppsala (population of approximately 170 000). The Fyrisån flows through the city just before reaching Lake Mälaren.

The Słupia catchment (1623 km^2^) is a coastal river basin in northern Poland. It drains into the southern Baltic Sea through the 138 km long Słupia River (Fig. 1). Climatically it belongs to *Dfb* class (warm-summer humid continental). Mean annual precipitation is 850 mm a^−1^ and mean (2000–2016) water flow at the outlet of the catchment is 17 m^3^ s^−1^, ranging from 8.4 to 53 m^3^ s^−1^. The relief is diverse and characterised by a mosaic of several morainic uplands and sandy outwash plains dissected with a network of eroding and tunnel valleys. The altitude ranges between 0 and 267 m a.s.l., with the highest parts in the south-east of the basin. The most typical soil types are sands and loamy sand. The middle part of the catchment is mostly covered by forest, with a considerable share of urban areas, while agricultural land is predominant in the southern and northern parts. Agricultural land and forest represent 49% and 44% of the catchment, respectively. Urban areas have been growing in size over recent years, and currently constitute approximately 5%, in majority covered by the city of Słupsk (approximately, 90 000 inhabitants).

### Identification of similar coastal catchments

Water quality modelling efforts often focus on small areas, predominantly on single catchments due to both systematic (catchment as the basic hydrological unit) and practical (data availability, time and labour input) reasons. On the other hand, regional studies are of great importance, especially in the case of evaluation of impacts related to policy actions (e.g. WFD or BSAP). Our study, covering three middle-sized coastal catchments in three different countries of the BSR, exemplifies such a situation. As in many other hydrological ‘case studies’, this raises the question: to which other geographical areas could conclusions of this study be transferred? One possible way of addressing this question is through identification of catchments that are ‘similar’ to those investigated in the present study. Catchment similarity can be analysed from different perspectives. Here we are mainly interested in factors affecting nutrient load generation process.

It should be emphasised that catchments geographically neighbouring on the three analysed catchments do not necessarily have to share similar behaviours and responses (He et al. 2011). This study adopted a catchment regionalisation perspective where three selected catchments serve as ‘donors’, and the goal is to derive a set of ‘target’ catchments based on certain similarity principles (McIntyre et al. 2005; Zhang and Chiew 2009). More specifically, we used the hydrological distance approach proposed by He et al. (2011). It had been previously applied for another purpose (model parameter transfer problem) in two large river basins in BSR, namely the Vistula and Odra by Piniewski et al. (2017). The method employs a set of physiographic characteristics to evaluate the physical similarity of catchments. In this study, the method was applied in a consistent manner, but separately for each country.

The analysis was based on four groups of catchment characteristics related to land use (percent of different types: urban, agriculture, forest, wetlands, and waters), soils (percent of gravel, sand, silt, and clay in topsoil and in subsoil, and percent of peat areas), topography (slope of agricultural land), and size. This set of characteristics is believed to describe the underlying physical processes leading to nutrient load generation from the landscape. We intentionally ignored other potential characteristics that are related to human activities, e.g. fertilizer rates, wastewater treatment plant (WWTP) loads and dams.

All characteristics were calculated by means of the available GIS data for a group of coastal catchments in each country. We excluded catchments smaller than 300 km^2^ and larger than three-fold area of a given catchment. We also focused on catchments draining directly to the Baltic Proper, Gulf of Finland, and Bothnian Bay. Climate was also taken into account by restricting the analysis to the *Dfb* zone, represented by all the catchments.

### SWAT model

SWAT is a process-based, semi-distributed, continuous-time model that simulates the movement of water, sediment, N and P compounds within a catchment with a daily time step (Arnold et al. 1998). SWAT requires specific information on weather, soil, topography, vegetation, and land management, and computes the processes associated with water and sediment movement, plant growth, nutrient cycling, etc. based on such input data. SWAT uses basic spatial units called HRUs (hydrological response units) combining land use, soil, and slope within each sub-basin. The land-phase total suspended solid (TSS) and nutrient transport components, namely land erosion, nutrient fluxes and water balance, are computed separately for each HRU, aggregated at the sub-basin level, and then routed through the river network to the main outlet.

A SWAT model application was developed for each catchment. The sources of data used in modelling of the three case study catchments are presented in Table S1 of the Electronic Supplementary Material (ESM). Despite some obvious differences in terms of data sources between countries, an attempt was made to develop SWAT models for each catchment in a consistent way. The catchments were delineated into 93, 51, and 76 sub-catchments, and 1708, 1760 and 1764 HRUs for Fyrisån, Vantaanjoki, and Słupia, respectively. Rigorous model calibration and validation for river discharge, sediment, and nutrient loads was performed for each case (see Piniewski et al. 2019 for the Vantaanjoki case). Basic characteristics of flow gauges and water quality monitoring points can be found in Table S2 (cf. Figure 1 for their location). Automatic calibration and uncertainty analysis with SWAT-CUP software (SUFI-2 programme) were carried out (Abbaspour et al. 2004). Kling–Gupta efficiency (KGE) number was adopted as the goodness-of-fit criterion (Gupta et al. 2009). However, we considered other important model performance measures such as R^2^ and percent bias (PBIAS) (cf. Table S6). The calibrated/validated model applications were used as ‘Baseline’ scenarios.

### Trends in the catchments

Collecting historical data were necessary for the analysis of current trends in the catchments’ characteristics influencing water quality, and for the development of a model scenario that would include near-future developments in the catchments. The historical data were collected in 6 thematic areas: *land use*, *agricultural area*, *livestock*, *fertiliser use*, *WWTP loads,* and *demography* (Table S2). We collected data at a spatial scale feasible for a given feature, ranging from catchments to administrative units such as municipalities, counties, provinces, or even countries. Although we used the data sources that to our knowledge are the best for each case, we acknowledge the fact that differences in spatial levels of aggregation may have an impact on trend analysis, particularly in the case when data for a larger unit (e.g. a province in Poland or a production area in Sweden) are taken to estimate trends in a catchment that occupies its small fraction. Annual data for the longest available sub-period within the period 2000-2018 were acquired for all characteristics except *land use*. For *land use,* we used the Corine Land Cover data base for years 2000, 2006, 2012, and 2018.

### Assessment of RBMPs

RBMPs are the basic planning documents required by WFD and the national Water Law for designing and enforcing actions aimed at the improvement or maintenance of good status of water bodies. The formal way of RBMP design and implementation is the discretion of Water Authority of a given Member State, but in all cases, the design of the Programme of Measures (PoM) requires the identification of the gap between the water’s current status and good status, followed by planning of a possibly cost-effective combination of measures.

Due to the differences in the RBMP development process in different countries, we collected the related country-specific information in Table S3. We selected measures feasible for SWAT modelling from each RBMP. In short, the Finnish RBMP contains primarily agri-environmental measures: buffer zones, constructed wetlands, winter time vegetation cover of fields, and perennial grass cultivation. The Swedish plans include the same first two measures as the Finnish ones, followed by reduced P leakage from spreading of manure, and stormwater ponds in urban areas. In contrast, the Polish RBMP only includes measures related to the wastewater sector: WWTPs modernization/extension, constructing new on-site WWTPs, and constructing new septic tanks.

### Targeting of measures

WFD implementation has triggered a shift towards a targeted approach to the placement of measures that could significantly improve their environmental efficiency and cost efficiency (Doody et al., 2012). As revealed by our analysis of RBMPs, they often specify the quantity or spatial extent of particular measures (e.g. X constructed wetlands, Y ha of buffer zones), but rarely their precise location within the river basin. In Finland, targeting of measures is recommended in the current programming period, but in practice it has remained rather limited.

From the modelling perspective, the placement of measures is one of the key decisions in scenario design. In the case of no evidence or suggestions regarding the placement of measures, they can be placed randomly. Another approach is spatial targeting of measures at certain areas within the model setup (in the case of SWAT they would be sub-basins or HRUs). Here, we have applied simple common-sense rules towards targeting, summarised as follows:

- winter time vegetation cover and buffer zones are targeted at agricultural HRUs with highest slopes;
- constructed wetlands are primarily targeted at sub-basins with a high proportion of agricultural land;
- stormwater ponds are targeted at sub-basins with a high proportion of urban land;

### Implementation of scenarios in SWAT

This study involved the development of a consistent set of four scenarios per catchment: (i) Baseline, describing the current status of nutrient loads (years 2002–2016) based on calibrated models, and three scenarios looking into the mid-term (~ 15 year) development of nutrient loads: (ii) Business-As-Usual (BAU), extrapolating historical trends in key variables affecting water quality into the future, (iii) BAU + RBMP, adding currently implemented and recommended measures to achieve the WFD goals for pollution reduction to BAU scenario, and (iv) BAU + RBMP + targ, either adding the same measures that were already planned under RBMPs, but targeting them at potential nutrient load generation hot spots, as described in “Targeting of measures WFD” section (Vantaanjoki and Fyrisån), or adding new measures (from outside RBMPs) and also targeting them at hot spot areas (Słupia).

Table 2 shows how particular factors of BAU scenario and measures included in BAU + RBMP and BAU + RBMP + targ scenarios were implemented in SWAT. For BAU scenario, the situation was straightforward—all three factors, i.e. land use change, fertilisation change and change in load from WWTPs, were analysed in each catchment. Measures included in RBMPs, however, differed between catchments, and so did the implementation of these measures in SWAT.

**Table 2:** Implementation of scenario factors and measures in SWAT for three studied catchments

Factor/measure	SWAT implementation	Scenario	Catchment
Land cover change	Parameters controlling hru size in.hru files and urban.dat file	BAU	Fyrisån, Vantaanjoki, Słupia
Fertilization change	Mineral fertilizer or manure application rates in.mgt files	BAU	Fyrisån, Vantaanjoki, Słupia
Change in load from WWTPs	Nutrient load values in point source files representing WWTPs	BAU	Fyrisån, Vantaanjoki, Słupia
Buffer zones	Vegetative filter strip function in.ops files	BAU + RBMP, BAU + RBMP + targ	Fyrisån, Vantaanjoki,
Constructed wetlands	Wetland parameters in.pnd files	BAU + RBMP, BAU + RBMP + targ	Fyrisån, Vantaanjoki, Słupia
Wintertime vegetation cover of fields	Management practices in.mgt files	BAU + RBMP, BAU + RBMP + targ	Fyrisån, Vantaanjoki, Słupia
Perennial grass cultivation	Management practices in.mgt files	BAU + RBMP	Vantaanjoki
Reduced phosphorus leakage from spreading of manure	Fertilizer operation parameter in.mgt files	BAU + RBMP, BAU + RBMP + targ	Fyrisån, Vantaanjoki, Słupia
Stormwater ponds in urban areas	Pond parameters in.pnd files	BAU + RBMP, BAU + RBMP + targ	Fyrisån, Słupia
WWTPs modernization/extension	Nutrient load values in point source files representing WWTPs	BAU + RBMP	Słupia
Constructing new on-site WWTPs	Nutrient load values in point source files representing on-site WWTPs	BAU + RBMP	Słupia
Constructing new septic tanks	Nutrient load values in point source files representing septic tanks	BAU + RBMP	Słupia

*BAU* Business-As-Usual, *RBMP* River Basin Management Plans, *targ* targeted measures, *WWTPs* Wastewater Treatment Plants

## Results

### Identified similar coastal catchments

**Fig. 2:**
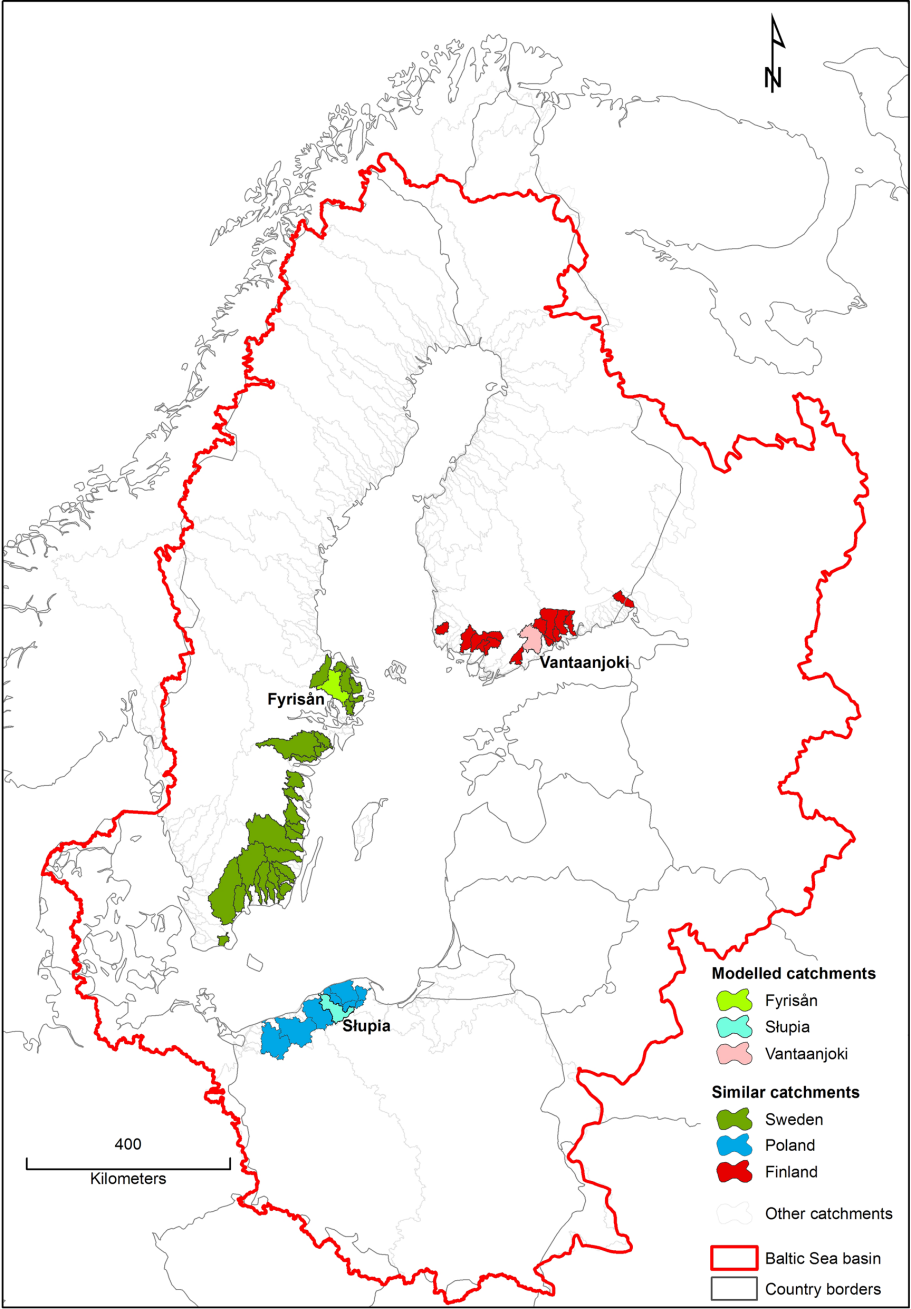
Location of the identified coastal catchments that are ‘similar’ to three catchments modelled in this study

The application of the hydrological distance approach described in “Identification of similar coastal catchments” section resulted in the selection of 28 Swedish coastal catchments covering an area of 32 000 km^2^ similar to Fyrisån, 12 Finnish coastal catchments covering an area of 8 200 km^2^ similar to Vantaanjoki, and six Polish coastal catchments covering an area of 11 000 km^2^ similar to Słupia (Fig. 2). A high number of similar catchments derived for Sweden resulted from fairly uniform values of catchment characteristics calculated for Swedish catchments. In the case of Finland, the situation was different due to two reasons: high fraction of urban land cover in Vantaanjoki, and high fraction of peat soils present in northern Finland. Only nine catchments in Poland met the initial criteria for the analysis, primarily due to much shorter coastline.

The total area of which the three selected catchments are representative is almost ten times greater than the total area of the three catchments themselves.

### Model calibration and validation

Calibration and validation for mean flow (m^3^ s^−1^) as well as sediment (t day^−1^) and nutrient (kg day^−1^) loads were performed for each of the study catchments. For flow, daily mean values were used in all three catchments. In terms of sediment and nutrient loads, in Vantaanjoki and Fyrisån daily mean values were used, and in Słupia monthly mean values. In Vantaanjoki, the daily load dataset was based on continuous (hourly) water quality and daily flow datasets (see Piniewski et al. 2019), and in Fyrisån we computed daily loads only for days when concentration data were available (typically once per month) and used them as the observational data set for calibration.

**Fig. 3:**
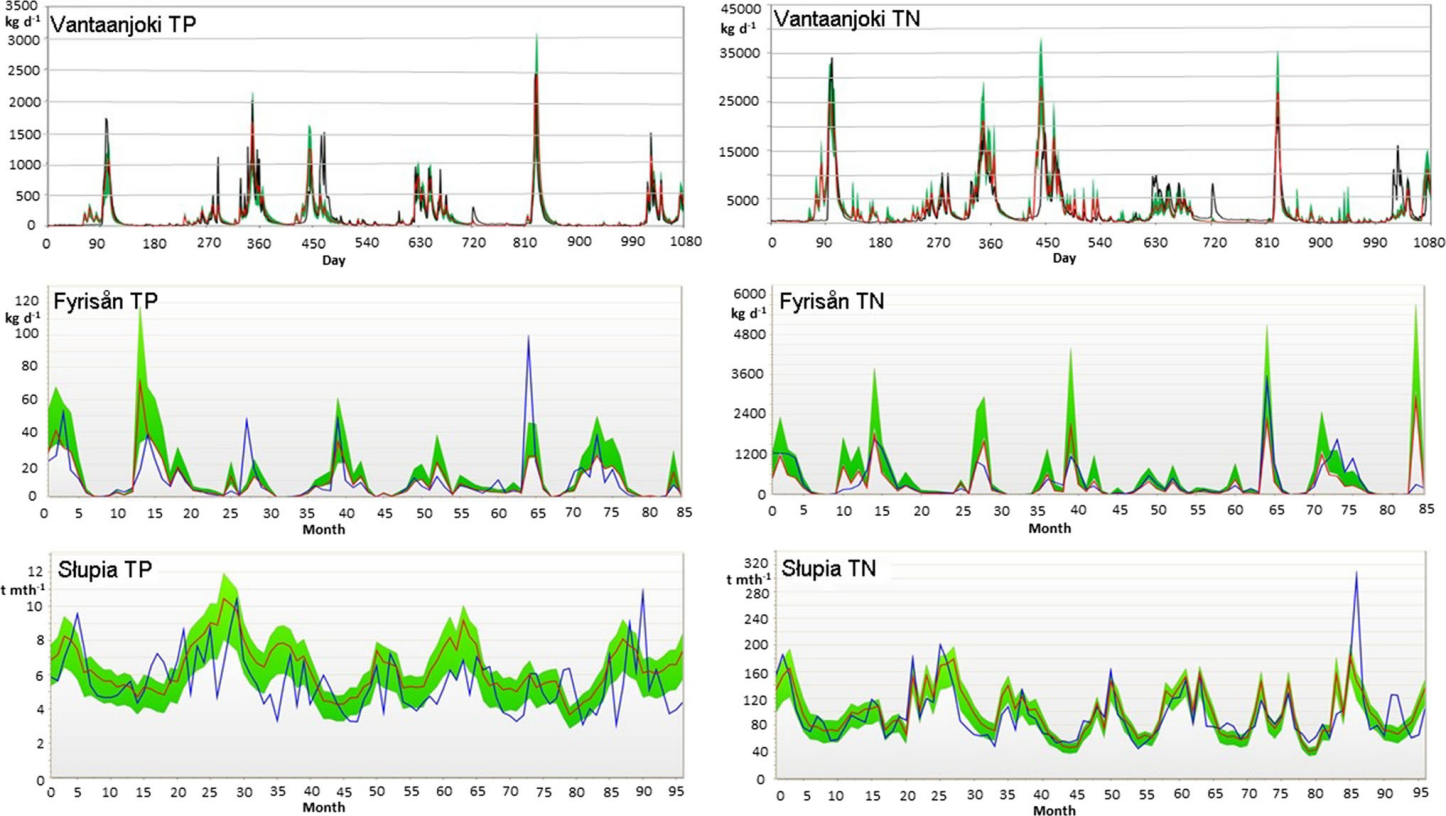
Modelled (red) and observed (blue) total phosphorus (TP) and total nitrogen (TN) loads (kg day^−1^, except for Słupia t month^−1^) in the case study catchments for the calibration periods (see Table 3), The green areas describe 95% prediction uncertainty

KGE values for flow shown in Table 3 can be generally regarded as high (in addition, the values of R^2^ and percent bias, PBIAS, are provided in Table S2). Therefore, we can rely on SWAT applications to accurately simulate the hydrology of the catchments. The best results for nutrients were achieved for Vantaanjoki (for both calibration and validation periods) (see Piniewski et al. 2019). This is also reflected in similar temporal dynamics between the observed and simulated values during the calibration periods (Fig. 3). For Fyrisån the KGE values for total nitrogen (TN) and total phosphorus (TP) were slightly lower than for Vantaanjoki, but still good or satisfactory. As presented in Fig. 3, the variability of nutrient loads was incomparably lower than for Vantaanjoki, potentially resulting from two aspects: (1) scarce observational data set that did not capture many peak events; (2) more flashy character of Vantaanjoki. In Słupia, the KGE values of calibration can be assessed as good for TN and satisfactory for TP and TSS. The validation results for TP, however, are relatively low. It should be noted that for Słupia, calibration and validation were performed only for monthly data. The temporal variability of monthly loads in Słupia was incomparably lower than that of the daily loads in Vantaanjoki, and even in Fyrisån. This can be explained by the difference in time scale, as well as stable flow regime related to the buffering effect of lakes and permeable soils (Fig. 3). Significantly lower model performance for TP compared to TN in Polish conditions was also reported by other authors (Ostojski et al., 2014; Marcinkowski et al., 2013). Despite low correlation between simulated and observed monthly TP loads, the long-term mean load was accurately captured in Słupia, with most observations included in the 95% prediction uncertainty band (Fig. 3).

**Table 3:** Kling–Gupta (KGE, Gupta et al. 2009) goodness-of-fit numbers for SWAT calibration and validation for the observed values of sediment (TSS), total phosphorus (TP) and total nitrogen (TN) loading. The optimal KGE value is 1

Catchment	Period	Flow*	TSS	TP	TN
Vantaanjoki**	Calibration 2011–2013	0.88	0.79	0.83	0.76
	Validation 2014–2016	0.86	0.73	0.69	0.72
Fyrisån	Calibration 2001–2007	0.88/0.74	0.65	0.62	0.75
	Validation 2008–2016	0.83/0.66	0.63	0.45	0.70
Słupia	Calibration 2000–2007	0.76	0.55	0.44	0.71
	Validation 2010–2015	0.85	–***	0.29	0.69

*Vantaanjoki calibration period 2003–2008 and validation period 2009–2016, Słupia calibration period 2001–2006 and validation period 2007–2016, Fyrisån calibration period 2001–2007 and validation period 2008–2015. Fyrisån KGE values represent northern/eastern parts of the catchment

**Load calibration/validation against continuous data (see Piniewski et al. 2019)

***Validation for TSS was not performed due to missing data

The resulting modelled baseline nutrient loads for the case study catchments are as follows: 0.31, 0.18, and 0.44 kg P ha^−1^year^−1^ and 6.8, 7.0, and 7.8 kg N ha^−1^year^−1^ for Vantaanjoki, Fyrisån, and Słupia, respectively. The results suggest that the highest share of agriculture in Słupia is reflected by the highest specific nutrient load. The difference between Vantaanjoki and Fyrisån regarding TP is in line with the general trend of specific TP load into the Baltic Sea; lower from Sweden than from Finland (HELCOM 2018). The multi-annual variability of nutrient loads is the highest for Vantaanjoki for both TN and TP.

### Trends in factors affecting water quality

**Fig. 4:**
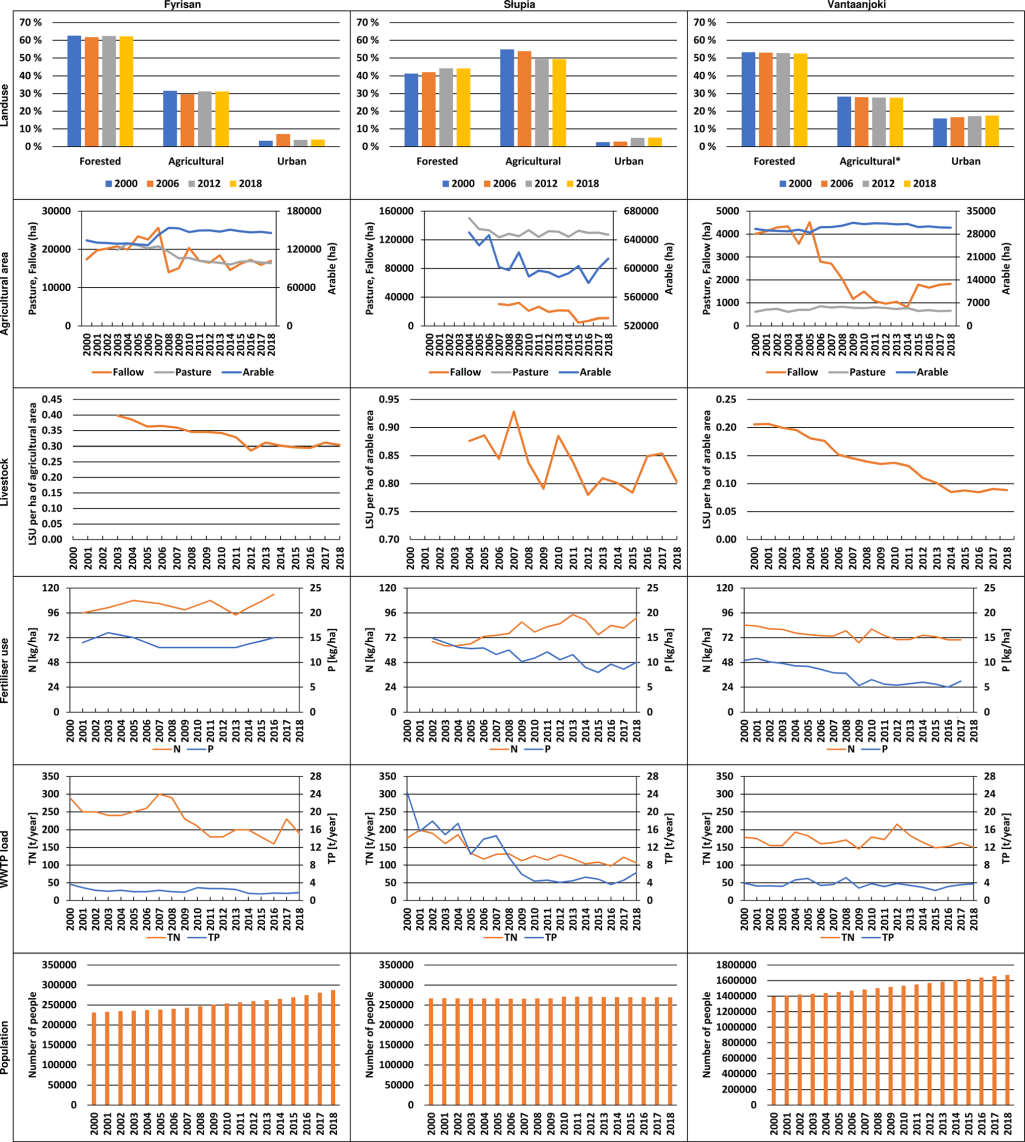
Historical trends in selected key factors in studied catchments

Figure 4 presents historical trends for key factors affecting water quality in the analysed catchments. While some factors are observed to undergo rapid changes (e.g. WWTP loads and livestock density), others are subject to small to moderate changes (agricultural area, fertiliser use), or remain fairly stable during the analysed period (land cover). In most cases, the patterns were consistent across catchments, but in some cases, such as N mineral fertiliser use, small differences occurred (a small increase in N use in Poland and Sweden compared to a small decrease in Finland). A more detailed discussion about the analysed trends is included in Table S4. For BAU scenario, we used the historical trends presented in Figure 4 as input for developing its assumptions for the SWAT model runs. The trends, if present, were extrapolated into near future. The analysis of RBMPs presented in  “Assessment of RBMPs” section allowed for developing assumptions for a scenario called BAU + RBMP. In this scenario, measures were placed randomly without any spatial targeting. In the final step, we developed scenarios (called BAU + RBMP + targ) in which measures were targeted at the areas considered hot spots for N and P emission in each catchment. A summary of all scenario assumptions is presented in Table 4.

**Table 4:** Assumptions for the BAU scenario based on the analysis of historical trends and RBMPs for each catchment

Catchment	Scenario BAU		Scenario BAU + RBMP		Scenario BAU + RBMP + targeting	
	Factor	Extent	Measure	Extent	Measure	Extent
Vantaanjoki	Land cover changes	Urban + 3%; Forest − 1%; Pasture − 2%	Buffer zones	360 ha	Buffer zones	Targeted
	Fertilizers	Mineral N − 10%; Mineral P − 40%; Manure 40%	Constructed wetlands	36 pcs.	Constructed wetlands	Targeted
	Nutrient loads from WWTPs	TN − 10%; TP − 20%	Wintertime vegetation cover of fields (70% winter stubble; 30% direct sowing)	13500 ha	Wintertime vegetation cover of fields	Targeted
			Perennial grass cultivation	4824 ha		
Fyrisån	Land cover changes	Urban + T1%; Fallow land − 1%	Buffer zones	55 ha	Buffer zones	Targeted
	Fertilizers	Mineral N +10%; Mineral P 0%; Manure − 20%	Constructed wetlands	50 ha	Constructed wetlands	Ttargeted
	Nutrient loads from WWTPs	TN -20%; TP-25%	Reduced phosphorus leakage from spreading of manure	Manure application areas		
			Stormwater ponds in urban areas	25 ha	Stormwater ponds in urban areas	
Słupia	Land cover changes	Forest + 3%; Fallow land − 3%	WWTPs modernization/extension	5 pcs.	Wintertime vegetation cover of fields	Targeted
	Fertilizers	Mineral N + 25%; Mineral P − 30%; Manure − 15%	Constructing new on-site WWTPs	1104 pcs.	Reduced phosphorus leakage from spreading of manure	Manure application areas
	Nutrient loads from WWTPs	TN -20%; TP-35%	Constructing new septic tanks	340 pcs.	Slowing down emissions from drainage systems	Targeted
					Stormwater ponds in urban areas	

### Model-based assessment of future nutrient loads

**Fig. 5:**
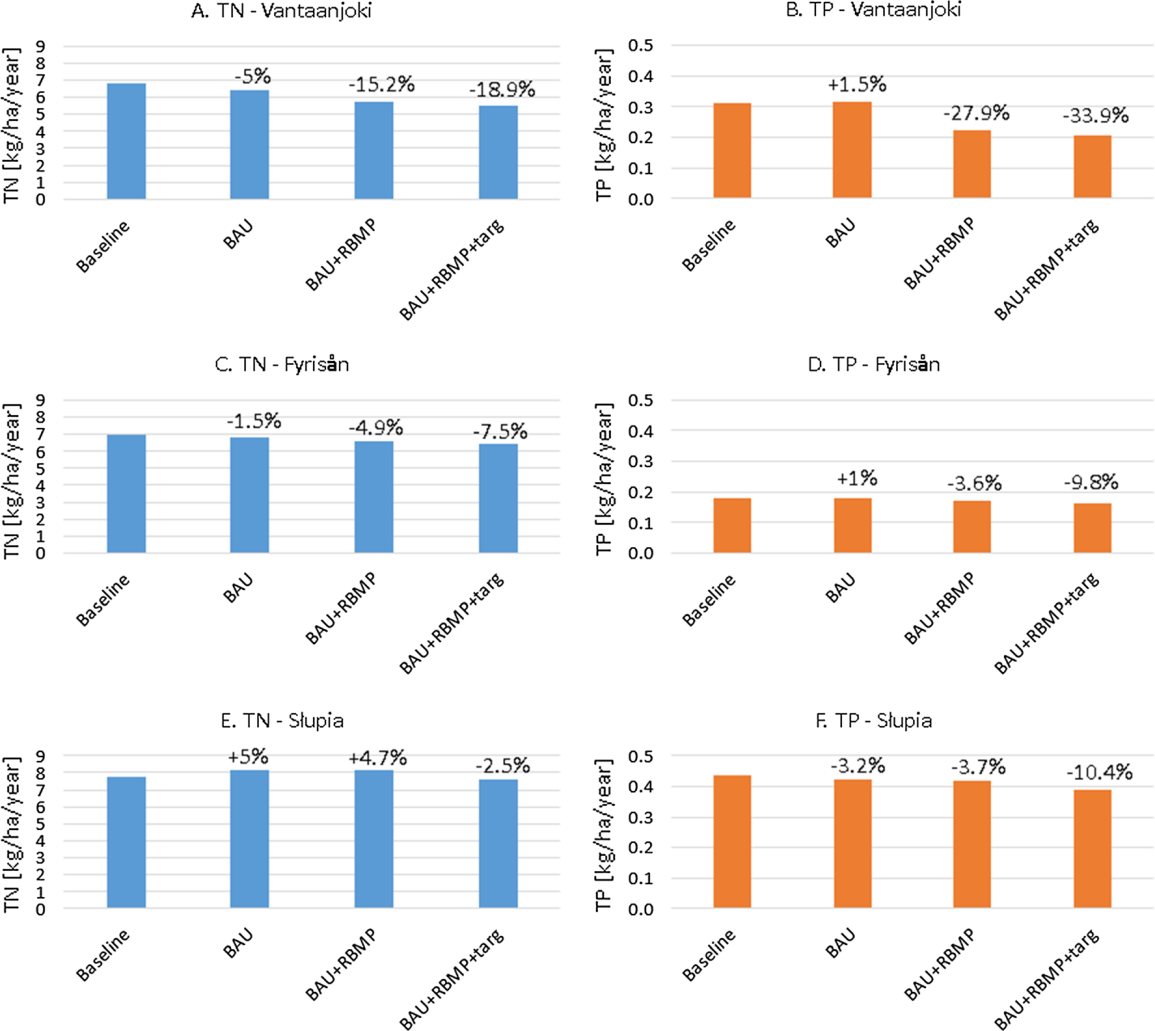
Mean annual area-specific loads of TN and TP from case study catchments in different scenarios

As shown in Fig. 5, only modest changes in TN and TP loads are predicted for BAU scenario for all the analysed catchments. The smallest changes, not exceeding 2% in absolute values, occur in Fyrisån. The changes for Vantaanjoki and Słupia show two opposite directions for TN and TP. In Słupia, TN loads increase by 5%, whereas in Vantaanjoki they decrease by 5%. This difference is primarily caused by a difference in N fertiliser change (Table 4). For TP loads, a minor increase by 1.5% is observed for Vantaanjoki, and a decrease by 3.2% for Słupia. This change is predicted to happen in response to reduced fertiliser rates and TP loads from WWTPs.

BAU + RBMP scenario brings quite different conclusions for each catchment. It has a clearly negligible effect in Slupia (decreases by less than 0.5% compared to BAU, cf. Figure 5), a moderate effect in Fyrisån (net decreases below 5%), and a much stronger effect in Vantaanjoki, reaching almost 30% for TP and 10% for TN (in comparison to BAU). This is in accordance with the assumptions and scope of this scenario presented in Table 4. Although measures planned in Finnish and Swedish RBMPs were fairly similar, their spatial extent was much greater in Vantaanjoki, resulting in higher effectiveness, particularly for TP. Additional analysis based on decomposition of BAU + RBMP scenario for Vantaanjoki pointed to wintertime vegetation cover on fields as the most effective measure that contributed the most to the overall “success” of RBMP (Fig. S1).

It is noteworthy that while changes in nutrient loads were highly variable across catchments for BAU + RBMP scenario, they were much more homogenous for BAU + RBMP + targ scenario. Targeting of measures was effective in each catchment, further decreasing the reduction of TN and TP loads by several percent. In Vantaanjoki and Fyrisån, the additional reductions due to targeting were twice as high for TP than for TN loads, whereas for Słupia net differences were relatively similar for both parameters (7–8%). It should be emphasised, however, that in the case of TN, the precicted loads for BAU + RBMP were higher than for the Baseline scenario, and targeting contributed to its reduction.

**Fig. 6:**
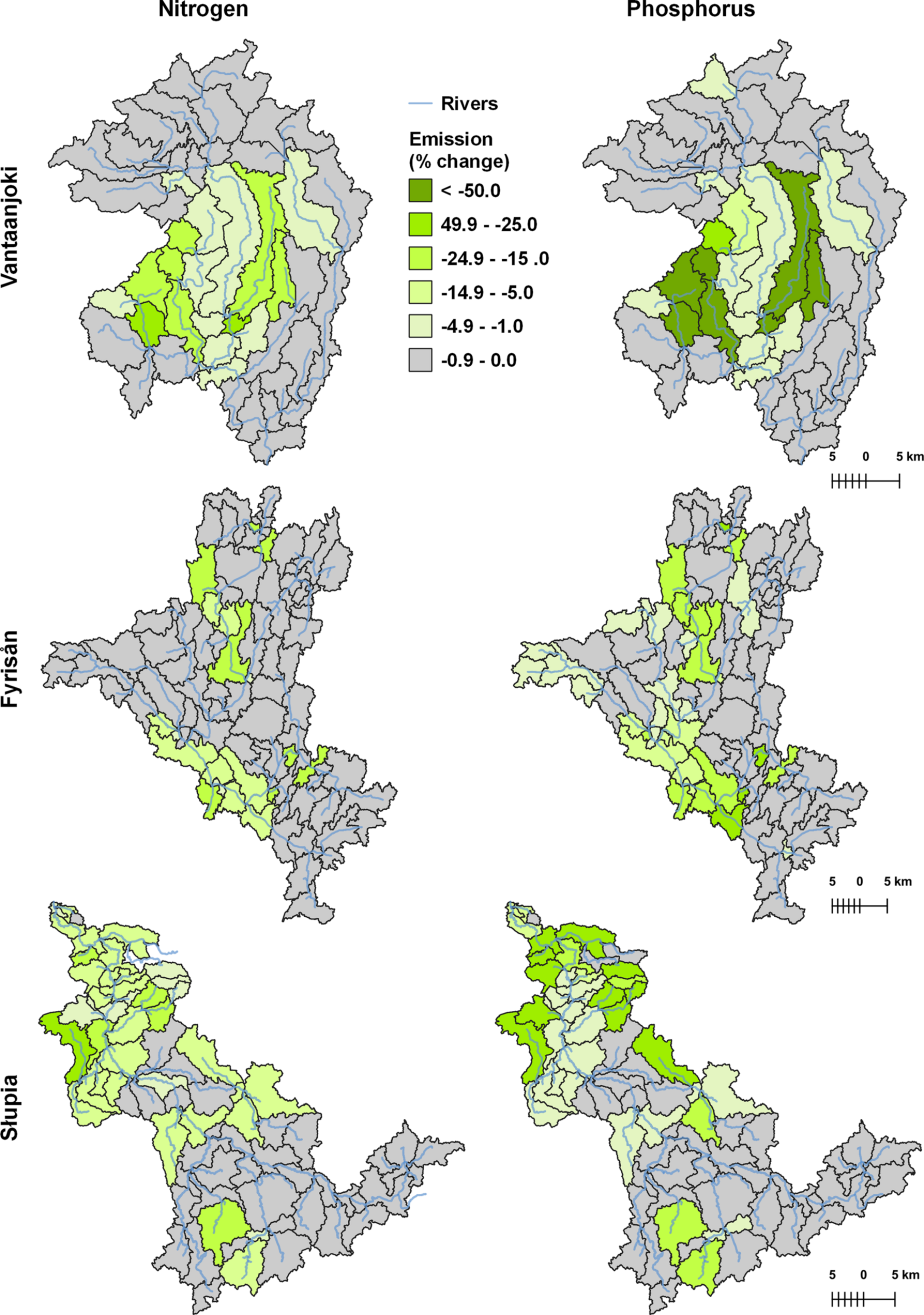
Average percent decrease in TN and TP emission in BAU + RBMP + targ scenario as compared to BAU + RBMP scenario at subbasin level for three analysed catchments

While the results discussed so far represent the stiuation observed at the outlets of three catchments, the spatial patterns within each catchment are also interesting. Figure 6 shows the effectiveness of targeting applied in BAU + RBMP + targ scenario compared with BAU + RBMP scenario for both TN and TP emission at SWAT sub-basin level. Not surprisingly, the “local”, sub-basin scale effects of targeted measures can be much higher than the effects simulated at the main outlets. The highest reduction exceeding 50% was found for TP in several sub-basins of the Vantaanjoki catchment. More detailed maps, also presenting mean annual loads generated in both scenarios as well as reductions expressed in absolute terms (kg ha^−1^year^−1^), are included in Figs. S2–S7.

## Discussion

Our modelling results show high variability across three BSR catchments, nutrients, and proposed scenarios. First, BAU scenario based on extrapolation of the existing socio-economic trends did not affect the modelled nutrient loads by more than 5% in terms of absolute values. This suggests that the impact of ongoing development remains relatively limited, and the system response could be expected to be more intensive due to management measures. The effectiveness of the existing RBMPs in nutrient load reduction, however, considerably varies across catchments. The boldest plans in terms of the spatial extent of measures seem to exist in Finland. If implemented, they would lead to a considerable reduction of TN (15%) and TP (28%) loads to the Gulf of Finland compared to the baseline. While the set of measures proposed in Swedish plans is partly similar to that in the Finnish ones, their spatial extent is much more modest, resulting in significantly lower reductions. The situation in Poland is different. The current RBMP only covers measures affecting the wastewater sector, and as shown in this study, their effect is almost negligible. Agricultural measures are clearly missing in this plan—point sources from WWTPs constitute only approximately 11% of TN and TP loads in the Słupia catchment (Koskiaho et al. 2020). On the other hand, a large part of Poland, a country in which about 40% of total agricultural land of the Baltic Sea catchment is located, is characterised by high P surpluses that are linked to high livestock densities (in Poland especially pig farms; Svanbäck et al. 2019), as well as low P use efficiency compared to other BSR countries except Russia and Belarus (McCrackin et al. 2018). This suggests that counteracting separation of crop and livestock production as well as improving manure use efficiency could be useful measures aimed at achieving nutrient reduction targets. This would, however, require a closer integration of the WFD and CAP (Common Agricultural Policy), which remains a challenge due to the fact that, monitoring and evaluation systems of CAP are of limited use from the water management perspective and there does not exist any other information system that could provide data required for linking agricultural practices with water quantity and quality (European Court of Auditors 2014). Lack of integration of the WFD and CAP is particularly noticeable in countries with a large share of agricultural land, such as Poland. Further progress in this respect can be achieved through consistent modelling of the effect of agriculture on water resources, as carried out in this study.

Adding spatially targeted measures to the existing RBMPs would considerably improve their effectiveness in all three catchments for both TN and TP. This is an important outcome that should encourage a wider adoption of targeted measures. Similar implications for TP were drawn in Finland by Puustinen et al. (2019). Simulated reductions, however, should be analysed in a wider context of HELCOM CARTs, as well as reduction targets imposed by the WFD (not existing in Poland—cf. Table 1). Despite a clear spatial discrepancy between country-scale reduction targets and medium-sized catchment scale in this study, our analysis showed that the selected catchments can be considered representative of much larger areas (cf. Figure 2). Assuming the binding character of the country-scale targets in the analysed catchments, it seems that under BAU + RBMP + targ scenario they can be achieved in Vantaanjoki for both TN and TP, and in Fyrisån for TN. They are unlikely to be achieved in Fyrisån for TP, or in Słupia for either TN or TP. It should be emphasised that in the latter case, the gap between the simulated and desired (CART) reduction is vast for TP: 10% vs. 61%. This points to the questionable feasibility of reduction targets for Poland pointed out in other studies (Pastuszak et al. 2018).

As all modelling studies, also this study has some limitations worth discussing. Not all measures proposed in RBMPs can be modelled with SWAT. Some of the measures are vaguely expressed and/or not quantifiable. In Finland, for example, the RBMP included measures entitled “Land application of slurry and enhanced reduction of nutrient load” and “Agri-environmental guidance” which we did not include in this study. “Structural liming” focused on the improvement of clayey soil structure, proposed in the Swedish RBMP, could also not be modelled.

According to the model simulation by Puustinen et al. (2019), measures implemented in the current RBMP period (2016-2021) have reduced the erosion of agricultural land by 17% and load of particulate P by 13% in Finland. As the winter time stubble, lighter tillage, and direct sowing area expanded, the leaching of dissolved reactive P (DRP) increased by 7% from its initial level. Due to this, the TP load reduction remains at 5%. As the area ploughed in autumn was halved, the calculated load of TN decreased by 13%. The possible reduction in the use of fertilisers was not taken into account in the simulation (Puustinen et al. 2019). It was found that leaching of DRP can be so high, e.g. as a result of the release of DRP from plant waste (e.g. Djodjic et al. 2000), that despite the reduced erosion, leaching of TP is increased (Uusitalo et al. 2018). No-till decreased TP losses by 27% compared with traditional autumn ploughing, corresponding well with the SWAT results of this study for the Vantaanjoki. At the same time, no-till increased DRP loss to an extent where particulate phosphorus (PP) loss decreased by even 54%. In this study, scenario BAU + RBMP brought a strong effect in Vantaanjoki, reaching almost 30% for TP and 10% for TN. Nitrogen and particle bound P is in line with previous national studies, but for TP, SWAT seems to overestimate the effect of the measures. This discrepancy could be partly explained by the fact that SWAT does not take into account the increase in soluble phosphorus caused by winter time vegetation cover.

This study focused on the mid-term future horizon reaching 15 years ahead and several factors undoubtedly affecting water resources in the BSR, but neglected the associated impacts of climate warming. This is primarily due to the assumption that such a short time horizon permits prioritising driving forces other than climate. The majority of recent climate impact studies in the region have been indeed reaching the mid- or end of the century. Despite inevitable uncertainty associated with climate projections, a consistent picture arises from a review of these studies: climate warming is expected to increase nutrient loads across BSR. A large-scale study employing the E-HYPE model (Bartosova et al. 2019) showed a significant increase in both P and N loads delivered to the Baltic Sea in response to the projected climate change (ensemble mean change by 14% and 8%, respectively). Projections based on the Fluxmaster regression model showed a 14% median increase in total inorganic nitrogen export from Sweden (2061-2090) due to climate change (Teutschbein et al. 2017). An even sharper increase in nitrogen loads in response to changing climate was simulated in different catchments in Poland using SWAT (Piniewski et al. 2014; Marcinkowski et al. 2017) as well as MODFLOW and MT3DMS (Olesen et al. 2019). For P loads, the projected increases were lower in the study of Marcinkowski et al. (2017), whereas in the other study of Piniewski et al. (2014) they reached 20% (PO4-P). The conclusion from these studies in the context of the present work is that, in the long run, climate change may jeopardise efforts to cut emissions across the entire BSR.

Water quality modelling involving the analysis of land use change impacts and pollution control measures is typically carried out for small to meso-scale catchments. Reaching the ultimate goal of HELCOM to combat Baltic Sea eutrophication and meeting the WFD objectives of achieving good water status would require scientific output at the national or regional scale. In contrast to the majority of other studies in BSR, our paper involves consistent modelling in three countries, as well as an identification of similar coastal catchments in which the results are likely to be comparable to those obtained in this study. This approach may be adopted in other regions of the world as a means of seeking of geographical areas to which study conclusions could be transferred. Nevertheless, the scale mismatch remains a challenge. One of the possible ways forward was recently sketched for N reduction by Refsgaard et al. (2019), who advocated the concurrent use of small-scale, physically-based models and process-based models operational at macro-scale within a spatially differentiated regulation framework. Another future pathway involves optimization-based targeting permitting valuation of trade-offs between water quality and agricultural production (Bostian et al. 2015).

## Conclusions

Our model-based evaluation of policy and management in three countries in the BSR permits drawing the following conclusions:

1. We observe relative stability or a slight decrease in major triggers for nutrients emission over the last 15 years, with the most considerable changes in the Polish catchment, and an evident shift towards an increase in its urban areas and population. Assuming similar trends for the next 15 years (BAU), no significant decrease in N and P loads form medium-sized catchments can be expected.
2. We observe an evident positive impact of the planned WFD measures on a decrease in loads from the analysed catchments, particularly for P. The highest decrease is predicted for Finland and Sweden, and lowest for Poland due to the lack of agricultural measures in RBMPs.
3. Targeting of measures is expected to bring additional reduction of nutrient loads in all catchments, suggesting that it should be adopted more widely in the Baltic Sea countries.
4. In an attempt to upscale the above study findings, we identified 28 Swedish coastal catchments covering an area of 32 000 km^2^ similar to Fyrisån, 12 Finnish coastal catchments covering an area of 8 200 km^2^ similar to Vantaanjoki, and six Polish coastal catchments covering an area of 11 000 km^2^ similar to Słupia. This step from a ‘case study’ scale towards a regional scale, within the coastal zone of three BSR countries, is particularly relevant from the policy perspective.
5. Our assessment of the River Basin Management Plans shows that agricultural measures were numerous in Sweden and Finland and missing only in Poland. This, together with promising results of the ‘targeting scenario’, calls for a stronger integration of the WFD and CAP, which is a prerequisite for mitigating harmful impact of agriculture on the status of rivers and the Baltic Sea.

## Electronic supplementary material

Below is the link to the electronic supplementary material.

Supplementary material 1 (PDF 2831 kb)
