# Supplementary material for: How effective are River Basin Management Plans in reaching the nutrient load reduction targets?
Source: Ambio. 2020 Sep 27;50(3):706–22. doi: 10.1007/s13280-020-01393-x (PMC7882664; doi:10.1007/s13280-020-01393-x)
Supplement: Supplementary file 1 — Supplementary material 1 (PDF 2831 kb) [file 13280_2020_1393_MOESM1_ESM.pdf]

Ambio

Electronic Supplementary Material

This supplementary material has not been peer reviewed.

Title: **How effective are River Basin Management Plans in reaching the nutrient load reduction targets? – A model-based analysis of three medium-sized Baltic Sea river basins in Finland, Sweden and Poland**

Table S1 Modelling data sources

General	**Catchment**	**Vantaanjoki**		
	Catchment size [km^2]	1688		
**Item**		**Source**	**Resolution / scale**	**Description**
Watershed delineation	DEM	MML (National Land Survey of Finland) SYKE (Finnish Environment Institute)	Grid size 10m x 10m, the accuracy of the elevation 1.4 m	Based on contour lines of MML's terrain database.
	River network	SYKE	1:250 000	Required for "burning in" streams
	Water use and transfer locations		1 hydropower plant with weir (Vanhankaupunginkoski) and a weir at the outlet of the regulated lake Tuusulanjärvi, maybe some minor fish ponds in the upper reaches	Required in order to modify placement of inlets and outlets in watershed delineation
	Lake/reservoir map	SYKE	1:250 000	Required in order to modify placement of inlets and outlets in watershed delineation
	Gauge stations locations	SYKE/Hertta	9 points	Required in order to modify placement of inlets and outlets in watershed delineation
	Point source locations	SYKE/Vahti	21 municipal, 11 industrial	Required in order to modify placement of inlets and outlets in watershed delineation
HRU delineation	Land cover map	CORINE Land Cover 2012	20m x 20m	Principal input data for land cover map. Needs reclassification to SWAT classes
	Soil map	GTK (Finnish Geological Survey) and SYKE	1:200 000	Principal input data for soil map. Needs reclassification to SWAT classes

**Weather data definition**	Precipitation data	FMI (Finnish Meteorological Institute)	3 stations	Recommended to interpolate precipitation from points to subbasins (stations outside are useful)
	Temperature data		3 stations	
	Wind speed data		1 station	
	Relative humidity data		3 stations	
	Solar radiation data		1 station	
**Land management**	Crop structure	TiKe (Information Centre of the Ministry of Agriculture and Forestry) and MAVI (Agency for Rural Affairs)	Field-block level data	Data from several years needed in order to define crop rotations
	Mineral fertilisers	MMM (Ministry of Agriculture & Forestry)	Nation-wide limit values of fertilization	Required to define fertiliser rates in management schedules
	Livestock / manure	TiKe and MAVI	Farm-level data	Required to define manure rates in management schedules
	Other practices (tillage)	TiKe and MAVI	Field-block level data	Required for definition of management schedules
	BMPs	MAVI	Nation-wide database, from which the BMPs located on the Vantaanjoki area can be extracted.	Useful to include in the model setup, otherwise there might be problems in calibration

**Water management**	Reservoirs	SYKE	Data for each object	Morphometric parameters, outflow release rules
	Fish ponds		Data for each object	Water uptake, water discharge
	Irrigation	LUKE (Natural Resources Institute Finland)	ELY-centre (province) -level statistics	Irrigated area, timing and rates
	Water withdrawals	Waterworks of the municipalities using the surface or groundwater of the catchment	Data for each object	Amount (monthly), source
	Wastewater treatment plants	Municipalities discharging their wastewaters into the river Vantaanjoki.	Data for each object	Effluent parameters (monthly, if not available annual)
**Ground water**	Hydrogeology maps			Ground water elevation contours (5 m interval) - not required but can be useful
**Channel**	Channel cross-sections			Useful to update default SWAT channel dimensions
**Soil properties**	Soil physical parameters	Literature	Data for each soil class in SWAT	One of the most critical parts of the model setup
	Soil chemical parameters			Measurements bi-annual (spring, autumn); samples from 3 depths (30, 60, 90cm); determination NO_3&NH_4 in soil and ground water
**Atmospheric deposition**	N and P deposition data	SYKE	stations inside and stations outside catchment	Concentrations/loads of N and P (dry and wet deposition). P data cannot be used as SWAT input

General	**Catchment**	**Fyrisån**		
	Catchment size [km^2]	1982		
**Item**		**Source**	Resolution / scale	**Description**
Watershed delineation	DEM	Lantmäteriet (Swedish mapping, cadastral and land registration authority)	2 m	Lidar-based
	River network	Lantmäteriet Property map HL		https://www.lantmateriet.se/globalassets/kartor-och-geografisk-information/kartor/produktbeskrivningar/eng/e_fastshmi.pdf
	Water use and transfer locations	SMHI (Swedish Meteorological and Hydrological Institute)		Required in order to modify placement of inlets and outlets in watershed delineation
	Lake/reservoir map	Lantmäteriet Property map MV		https://www.lantmateriet.se/globalassets/kartor-och-geografisk-information/kartor/produktbeskrivningar/eng/e_fastshmi.pdf
	Gauge stations locations	SLU (water quality) SMHI (water discharge)		http://miljodata.slu.se/mvm https://www.smhi.se/klimatdata/hydrologi/vattenforing
	Point source locations	SMED		http://tbv20.smhi.se/tbv/granska/
HRU delineation	Land cover map	Swedish Bord of Agriculture (SBoA)	Block data	Potential data source for improvement of land cover map in agricultural areas
	Soil map	Digital soil map	50 x 50 m	http://markdata.se/

**Weather data definition**	Precipitation data	SMHI		https://www.smhi.se/klimatdata/meteorologi/temperatur
	Temperature data			https://www.smhi.se/klimatdata/meteorologi/nederbord
	Wind speed data			https://www.smhi.se/klimatdata/meteorologi/vind
	Relative humidity data			
	Solar radiation data			https://www.smhi.se/klimatdata/meteorologi/stralning
**Land management**	Crop structure	SBo A	Block data	Data from several years needed in order to define crop rotations
	Mineral fertilisers	SCB (Statistics Sweden), SMED (Svenska MiljoEmissionsData)	Production areas level, production area 6 for Fyrisån	Required to define fertiliser rates in management schedules
	Livestock / manure	SCB, SMED	Production areas level, production area 6 for Fyrisån	Required to define manure rates in management schedules
	Other practices (tillage)	SCB, SMED	Production areas level, production area 6 for Fyrisån	Required for definition of management schedules
	BMPs	VISS		http://viss.lansstyrelsen.se/
**Water management**	Reservoirs	SMHI	Data for each object	Morphometric parameters, outflow release rules
	Fish ponds	SMED, SBoA	Data for each object	Water uptake, water discharge
	Irrigation	SCB, SBoA	Data for each object	Irrigated area, timing and rates
	Water withdrawals		Data for each object	Amount (monthly), source
	Wastewater treatment plants	Plant specific data, SMED	Data for each object	Effluent parameters (monthly, if not available annual)
**Ground water**	Hydrogeology maps	SGU (Geological Survey of Sweden)	https://www.sgu.se/en/groundwater/	Ground water elevation contours (5 m interval) - not required but can be useful

**Channel**	Channel cross-sections	SMHI		Useful to update default SWAT channel dimensions
**Soil properties**	Soil physical parameters	Literature	Data for each soil class in SWAT	One of the most critical parts of the model setup
	Soil chemical parameters	SMED, SLU, SBoA		Measurements bi-annual (spring, autumn); samples from 3 depths (30, 60, 90cm); determination NO_3&NH_4 in soil and ground water
**Atmospheric deposition**	N and P deposition data	SMHI		Concentrations/loads of N and P (dry and wet deposition). P data cannot be used as SWAT input

General	Catchment	**Słupia**		
	Catchment size [km^2]	1623		
**Item**		**Source**	Resolution / scale	**Description**
Watershed delineation	DEM	CODGIK (Centre for Geodetic and Cartographic Data)	10 m	Lidar-based
	River network	MPHP (Map of Hydrographic Division of Poland)	1:10 000	Required for "burning in" streams
	Water use and transfer locations	RZGW (Regional Water Management Authority) / WZMiUW (Land Reclamation Board)	5 small hydropower plants, 13 weirs, 11 objects drained areas, 17 fish ponds	Required in order to modify placement of inlets and outlets in watershed delineation
	Lake/reservoir map	MPHP (Map of Hydrographic Division of Poland)	1:10 000	Required in order to modify placement of inlets and outlets in watershed delineation
	Gauge stations locations	IMGW (Institute of Meteorology and Water Management)	8 points	Required in order to modify placement of inlets and outlets in watershed delineation
	Point source locations	RZGW (Regional Water Management Authority) / WIOŚ (Voivodship Institute of Environmental Protection)	9 municipal, 6 industrial	Required in order to modify placement of inlets and outlets in watershed delineation

**HRU delineation**	Land cover map	CORINE Land Cover 2012	The smallest polygon ~100 ha	Principal input data for land cover map. Needs reclassification to SWAT classes
		BDOT (Database of Topographic Objects)		Potential data source for improvement of land cover map in urban areas
		Copernicus Land Monitoring Service (Imperviousness 2012) http://land.copernicus.eu/pan-european/high-resolution-layers/imperviousness	20 m	Potential data source for improvement of land cover map in urban areas
		ODR (Agricultural Advisory Centres)	Commune level statistics on crop structure	Potential data source for improvement of land cover map in agricultural areas
	Soil map	WODGIK (Voivodship Centre for Geodetic and Cartographic Data), Forest department	1:2 000 -1:5 000	Too detailed map, needs reclassification
**Weather data definition**	Precipitation data	IMGW (Institute of Meteorology and Water Management)	11 stations (+36 stations outside of catchment)	Recommended to interpolate precipitation from points to subbasins (stations outside are useful)
	Temperature data		10 stations (some outside)	
	Wind speed data		2 stations	
	Relative humidity data		2 stations	
	Solar radiation data		1 station	To be acquired

**Land management**	Crop structure	ODR (Agricultural Advisory Centres)	Commune-level data	Data from several years needed in order to define crop rotations
	Mineral fertilisers	ODR (Agricultural Advisory Centres)	Commune-level data	Required to define fertiliser rates in management schedules
	Livestock / manure	ODR (Agricultural Advisory Centres)	Commune-level data	Required to define manure rates in management schedules
	Other practices (tillage)	ODR (Agricultural Advisory Centres)	Commune-level data	Required for definition of management schedules
	BMPs	ODR (Agricultural Advisory Centres)	Commune-level data	Useful to include in the model setup, otherwise there might be problems in calibration
**Water management**	Reservoirs	RZGW (Regional Water Management Authority)	Data for each object	Morphometric parameters, outflow release rules
	Fish ponds	RZGW (Regional Water Management Authority)	Data for each object	Water uptake, water discharge
	Irrigation	WZMiUW (Land Reclamation Board)	Data for each object	Irrigated area, timing and rates
	Water withdrawals	RZGW (Regional Water Management Authority)	Data for each object	Amount (monthly), source
	Wastewater treatment plants	WIOŚ (Voivodship Institute of Environmental Protection) + own survey	Data for each object	Effluent parameters (monthly, if not available annual)
**Groundwater**	Hydrogeology maps	PIG (Polish Hydrogeological Institute)	1:50 000	Ground water elevation contours (5 m interval) - not required but can be useful
**Channel**	Channel cross-sections	KZGW (National Water Management Authority)	One cross-section per 500 m on main rivers	Useful to update default SWAT channel dimensions

**Soil properties**	Soil physical parameters	Literature	Data for each soil class in SWAT	One of the most critical parts of the model setup
	Soil chemical parameters	OSChR (Chemical-Agricultural Stations)	21 locations	Measurements bi-annual (spring, autumn); samples from 3 depths (30, 60, 90cm); determination NO_3&NH_4 in soil and ground water
**Atmospheric deposition**	N and P deposition data	GIOŚ (Chief Inspectorate of Environmental Protection)	3 stations outside catchment	Concentrations/loads of N and P (dry and wet deposition). P data cannot be used as SWAT input

Table S2 Characteristics of flow gauges and water quality monitoring points used for calibration and validation in three catchments.

Catchment	River	Profile name	Upstream catchment area (km^2)	Sampling frequency	Data source*
		Flow gauges			
Fyrisån	Sävjaån	Sävja	722	daily	SMHI
	Fyrisån	Vattholma	294	daily	SMHI
Vantaanjoki	Vantaanjoki	Oulunkylä	1680	daily	SYKE
	Vantaanjoki	Myllymäki	1229	daily	SYKE
	Keravanjoki	Hanala	313	daily	SYKE
Słupia	Słupia	Sośnica	272	daily	IMGW-PIB
	Słupia	Charnowo	1558	daily	IMGW-PIB
	Skotawa	Skarszów Dolny	264	daily	IMGW-PIB
		Water quality monitoring points			
Fyrisån	Fyrisån	Vattholma N. Bron	294	monthly	SLU
	Fyrisån	Lena Kyrka	384	monthly	SLU
	Fyrisån	Vindbron	1254	monthly	SLU
	Sävjaån	Sävjaån Kuggebro	725	monthly	SLU
Vantaanjoki	Vantaanjoki	Pitkäkoski	1264	continuous	SYKE
Słupia	Słupia	Charnowo	1558	monthly	GIOŚ

* SMHI – Swedish Meteorological and Hydrological Institute; SYKE – Finnish Environmental Institute; IMGW-PIB – Institute of Meteorology and Water Management – National Research Institute; SLU – Swedish University of Life Sciences, Department of Aquatic Sciences and Assessment; GIOŚ – Chief Inspectorate for Environmental Protection.

Table S3 Sources and spatial level of aggregation of historical data used for trend analysis in three catchments.

Catchment	Data	Land use	Agricultural area	Livestock	Fertiliser use	WWTP loads	Demography
**Fyrisån**	level	catchment	county	field/block	production area	main plant	municipality
	source	CLC2000-2018	SBoA	SS	SS	UWW	SS
**Słupia**	level	catchment	province	province	province	county	county
	source	CLC2000-2018	SP	SP	SP	SP	SP
**Vantaanjoki**	level	catchment	municipality	municipality	country	catchment	county
	source	CLC2000-2018	LUKE	LUKE	SF	SYKE CMDS	SF

SBoA – Swedish Board of Agriculture, SS – Statistics Sweden, UWW – Uppsala Water and Waste, SP – Statistics Poland,

SF – Statistics Finland, LUKE – Natural Research Institute Finland statistical services,

SYKE CMDS - Compliance Monitoring Data System maintained by SYKE.

Table S4 Assessment of RBMPs

Finland
For water management planning, mainland Finland is divided into seven river basin districts, each of which has its own river basin management plan (RBMP). The ELY centers (Centre for Economic Development, Transport and the Environment) draw up a plan for their respective areas of activity. There are 13 ELY centers in Finland, so they must work closely together in drawing up the seven RBMP's. The Vantaanjoki watershed belongs to the Uusimaa ELY centre area and it covers 18% of the entire Uusimaa region. The Uusimaa ELY Center has convened a water management co-operation group representing the various stakeholders in the region as comprehensively as possible. The tasks of the co-operation group have been extended to include also coastal maritime management planning. Measures to improve water status have been sought as a step-by-step process. Actual data on the implemented agri-environmental mitigation measures are largely obtained centrally from the Finnish Food Authority. Quantitative data on the previous year's measures will be available yearly in May. Data are collected centrally and distributed by planning area.<br>In practice, agricultural measures have been implemented since 1995 through agri-environmental support schemes, to which almost 90% of farmers are committed. Due to the agri-environmental measures, e.g. the nutrient balances of the fields have decreased significantly, i.e. fertilization is applied according to the needs of the plants. Therefore, smaller amount of the nutrients is exposed to leaching after the growing season. Measures to increase the vegetation cover of fields during the winter, catch crops, as well as buffer strips and zones have also been able to reduce the risk of nutrient leaching. The use of these measures will be continued and further expanded through the RBMP's.<br>As the plans have been made to cover the entire Uusimaa region, the measures have been proportioned in relation to the areas of the Vantaanjoki catchment and the Uusimaa region. With regard to winter time vegetation cover, it is assumed that 70% of the fields are on stubble and 30% on direct sowing. The number of constructed wetlands proposed is 36, the implementation of which requires a great deal of effort and investment. The needed number of buffer zones is also large. In the previous planning period their number fell short of the target. Problems with the management of buffer zones and the complexity of the type of contract reduce farmers' interest in the measure. The area of perennial grassland is already rather high and if is significantly increased there should be means of circular economy to develop sustainable use of grass. In any case, grasslands should be located in areas where crop growth is weak, e.g. due to repeated flooding, or targeted as winter vegetation cover on sloping fields adjacent to water bodies.<br>There are also several ongoing projects in the Vantaanjoki river basin to study the effects of new measures such as the use of gypsum on water quality. However, these measures are not included in the current support scheme.
Sweden
Sweden is divided into five different water management districts, four of which are in the Baltic Sea Basin. For each of these districts, a county administrative board (*Länsstyrelse*) has been appointed to act as the water

authority (*Vattenmyndighet*) for the district in question. It is the water authority's responsibility that the Water Framework Directive (WFD) is implemented in the district in question. Consequently, it is also the water authorities that are responsible for preparation of management plans and action programs for the district in question (Vattenmyndigheterna 2020). The Fyrisån basin lies within the Northern Baltic Sea water district (*Norra Östersjöns Vattendistrikt)*.

The management plans and associated action programs are developed by water authorities in a process that also includes consultation with local authorities and other stakeholders. Stakeholder participation is commonly implemented via basin-level water boards or water councils (Jager et al. 2016). The actions included in the programs can largely be divided into two categories: *administrative actions* and *physical actions*. The former are directed at authorities (especially municipalities) that are given binding objectives to e.g., develop new rulesets or guidelines. The latter are actions that are implemented physically in the basin to enable fulfilment of the targets in the management plan. The physical actions in the action program are "proposed measures" in that they are not binding and that local authorities can choose to implement other measures that are found to be more effective or efficient (Vattenmyndigheten Norra Östersjön 2017).

Poland

In Poland, the National Water and Environmental Programme (NWEP) is a key and executive element of RBMPs. NWEP defines in practice basic and supplementary measures to be applied in pursuit to fulfil the WFD goals. As RBMPs, the NWEP undergoes 6 years planning cycle and is a subject to review and update in each cycle. The updated NWEP (planning cycle 2016-2021) as well as the database created within the programme were used to collect information on measures planned for the Słupia catchment. In total 121 individual, both basic and supplementary, measures were identified to be applied in 26 river and 11 lake water bodies in the catchment. They were grouped to four categories: (1) monitoring, (2) control measures, (3) municipal water management and (4) improvement of water regime and protection of water dependent ecosystems. It is noteworthy that no measures related to agricultural sector were identified for the catchment in the updated NWEP. Such measures were proposed in the NWEP for the previous planning cycle (2010-2015). We excluded from further analysis all measures that did not directly affect nutrient loads and water quality in rivers. As a result, only municipal water management measures were left: 83 individual measures that could be assigned to 5 following groups: building/modernization of sewage systems, modernization/extension of WWTPs, constructing on-site WWTP, constructing/repairing septic tanks and improvement of liquid disposal. Measures from the most of the groups were planned to be realized in majority of water bodies in the catchment. Only modernization/extension of WWTPs was restricted to only four water bodies, where obsoleted or insufficient WWTPs were located.

Table S5 Trends in factors affecting water quality

Land use
During the study period 2000–2018 in Vantaanjoki area there are slightly decreasing trends (below 1%) in forested and agricultural areas, while urban areas have increased by nearly 2%. In Fyrisån catchment the land use distribution was the most stable from all three. Changes for three major land use types were below 1%, with slight decrease in agricultural and forested areas, respectively by 0.4% and 0.3%, that was compensated by an increase in urban areas (0.7%). In Słupia catchment, two land use types (forested and urban areas) increased by more than 2%, which implies doubling the area for the latter. The increase in these two land use types was at the expense of agricultural areas that decreased by more than 5.5%. All of these changes were the most pronounced between 2000 and 2012.
Agricultural land
In Vantaanjoki, over the entire period, the area of arable land and pasture has been more or less constant with slight increase in the middle of the period. The fallow area decreased strongly during the whole period except for the last couple of years 2015–2018, when the fallow area have increased and arable land areas correspondingly decreased. In the Słupia catchment, all three agricultural land types, in general, have decreased over the entire period. In the case of arable land, the decrease reached its minimum in the year 2016 and then started to increase, but remain below 10% compared to the year 2004. Area of pasture consistently decreased between 2004-2007, and then stabilized with slight fluctuations from year to year. The area of fallow land consistently decreased, nearly three times until year 2015 and then started to slightly increase. In the case of the Fyrisån catchment, three major agricultural land use types behaved differently. Arable land was rather stable between 2000-2006, then increased within next three years until it stabilized, with a slight decreasing tendency for the rest of the period. Pasture area consistently decreased over the entire period, whereas fallow land area was fluctuating and at the end of the analysed period was similar to the area at the beginning.
Livestock
The LSU per arable land was the lowest in the Vantaanjoki area, and it strongly decreased until year 2014, reaching just 0.09 LSU/ha, after which it stabilized. LSU number in the Słupia catchment, despite fluctuating from year to year (range 0.78-0.93), was the highest overall. In general, a decreasing tendency was observed in the catchment. In the Fyrisån catchment LSU numbers showed clearly decreasing tendencies over the whole period. Similar to the Słupia catchment, the lowest LSU number was observed in 2012, and then it started to slightly increase, but still remaining below the initial number.

Fertilization
In the case of the Vantaanjoki catchment a marked decrease of both N and P fertilizer use was observed. Two regarded mineral fertilizers have undergone different changes in the Słupia catchment. In case of N there was a steady increasing tendency over the period of interest reaching over 90 kg ha^{-1} in 2018. For P, the tendency was the opposite. A decrease in use of P fertilizer was observed until 2015 followed by a slight increase (10 kg ha^{-1}). In the case of the Fyrisån catchment, the situation was the most stable among all three catchments. While N rates were slightly increasing, P rates could be considered as rather stable.
Demography
In terms of population in the Vantaanjoki catchment, there was a steady, year-to-year, increase by nearly 17% over the entire period. In the Słupia catchment the population change over the period of interest was slight, with an increase by 1%. Fyrisån catchment was very much like Vantaanjoki, and the data showed consistent, year-to-year, increase in population, reaching almost 20% over the entire period.
WWTP loads
N and P loads from WWTPs in the Vantaanjoki catchment were fluctuating during the studied period, but the fluctuation ranges were rather narrow and loads at the beginning and the end of the period were either the same (P) or slightly decreased (N). Despite the steady increase of number of people connected to WWTP over the whole period in the Słupia catchment, the total loads of both N and P, decreased during this time. The steepest decrease was observed in years 2000-2006 for N and in 2000-2009 for P. Afterwards, the situation stabilized, with annual fluctuations within a narrow range, and a slight increase in later years (2017-2018). In the Fyrisån catchment, for both N and P, a decreasing tendency in total loads was observed over the whole period. However, within the entire period, there were sub-periods of elevated loads, years 2005-2008 and 2013-2014 for N, and years 2010-2013 for P. Also, the later years (2017-2018) were characterized by slightly higher load values.

Table S6 Additional goodness-of-fit values for calibration and validation periods in three analysed catchments.

**Catchment**	**Period**	**Flow***		**TSS**		**TP**		**TN**	
		**R^2 (-)**	**PBIAS (%)**	**R^2 (-)**	**PBIAS (%)**	**R^2 (-)**	**PBIAS (%)**	**R^2 (-)**	**PBIAS (%)**
Vantaanjoki**	Calibration 2011–2013	0.79	1.3	0.69	-10.7	0.73	-7.8	0.68	10.1
	Validation 2014–2016	0.76	4.1	0.57	-10.4	0.5	1.2	0.58	2.0
Fyrisån	Calibration 2001–2007	0.78	-0.1	0.82	-14.6	0.75	-25.7	0.58	-27.7
	Validation 2008–2016	0.73	-4.0	0.37	-22.1	0.59	22.2	0.89	-17.6
Słupia	Calibration 2000–2007	0.65	-7.7	0.41	-8.9	0.23	-13.6	0.56	-4.2
	Validation 2010–2015	0.75	-1.8	***	***	0.15	-13.3	0.60	5.3

*Vantaanjoki calibration period 2003–2008 and validation period 2009–2016, Słupia calibration period 2001–2006 and validation period 2007–2016, Fyrisån calibration period 2001–2007 and validation period 2008–2015. Fyrisån KGE values represent northern/eastern parts of the catchment.

** Load calibration/validation against continuous data (see Piniewski et al. 2019)

*** Validation for TSS was not performed due to missing data.

Table S7 List of the most sensitive parameters for discharge, sediment, TN and TP loads in three catchments.

Parameter	Description	Sensitivity in catchment		
		Vantanjooki	Fyrisån	Slupia
	Discharge			
CN2	SCS Curve Number [–]	+	+	+
GW_DELAY	Groundwater delay [days]	+	+	
CNCOEF	Weighting coefficient used to calculate retention coefficient for CN [–]	+	+	+
SNOCOVMX	Minimum snow water content that corresponds to 100% snow cover [mm H_2O].		+	
SMTMP	Snow melt base temperature [°C].		+	
GW_REVAP	Groundwater revap coefficient [–]			+
ESCO	Soil evaporation compensation factor [–]			+
	Sediment			
PRF	Peak rate adjustment factor for sediment routing [–]	+	+	+
CH_COV1	Channel erodibility factor [–]	+		+
CH_COV2	Channel cover factor [–]			+
USLE_K	Soil erodibility factor	+	+	
RES_NSED	Equilibrium sediment concentration in the reservoir [mg/L]		+	
SPEXP	Exponent parameter for calculating sediment reentered in channel sediment routing [–]			+
	Nitrogen			
HLIFE_NGW	Half-life of nitrogen in groundwater [days]	+	+	
CDN	Denitrification exponential rate coefficient [–]	+	+	
NPERCO	Nitrogen percolation coefficient [–]	+		
SDNCO	Denitrification threshold water content [–]		+	
CMN	Rate factor for humus mineralization of active organic nutrients [–]		+	+
FRT_KG	Amount of fertilizer applied to HRU [$kgha^{-1}$]		+	+
SOL_ORGN	Initial organic N concentration in the soil layer [mg N kg^{-1}]			+
SOL_NO3	Initial NO_3 concentration in the soil layer [mg N kg^{-1}]			+
	Phosphorus			
ERORGP	Phosphorus enrichment ratio for loading with sediment [–]	+	+	+
PSP	Phosphorus availability index [–]	+	+	+
P_UPDIS	Phosphorus uptake distribution parameter [–]		+	
LAT_ORGP	Organic P in the base flow [mg L^{-1}]			+
GWSOLP	Concentration of soluble phosphorus in groundwater contribution to streamflow from sub-basin [mg P L^{-1}]		+	+

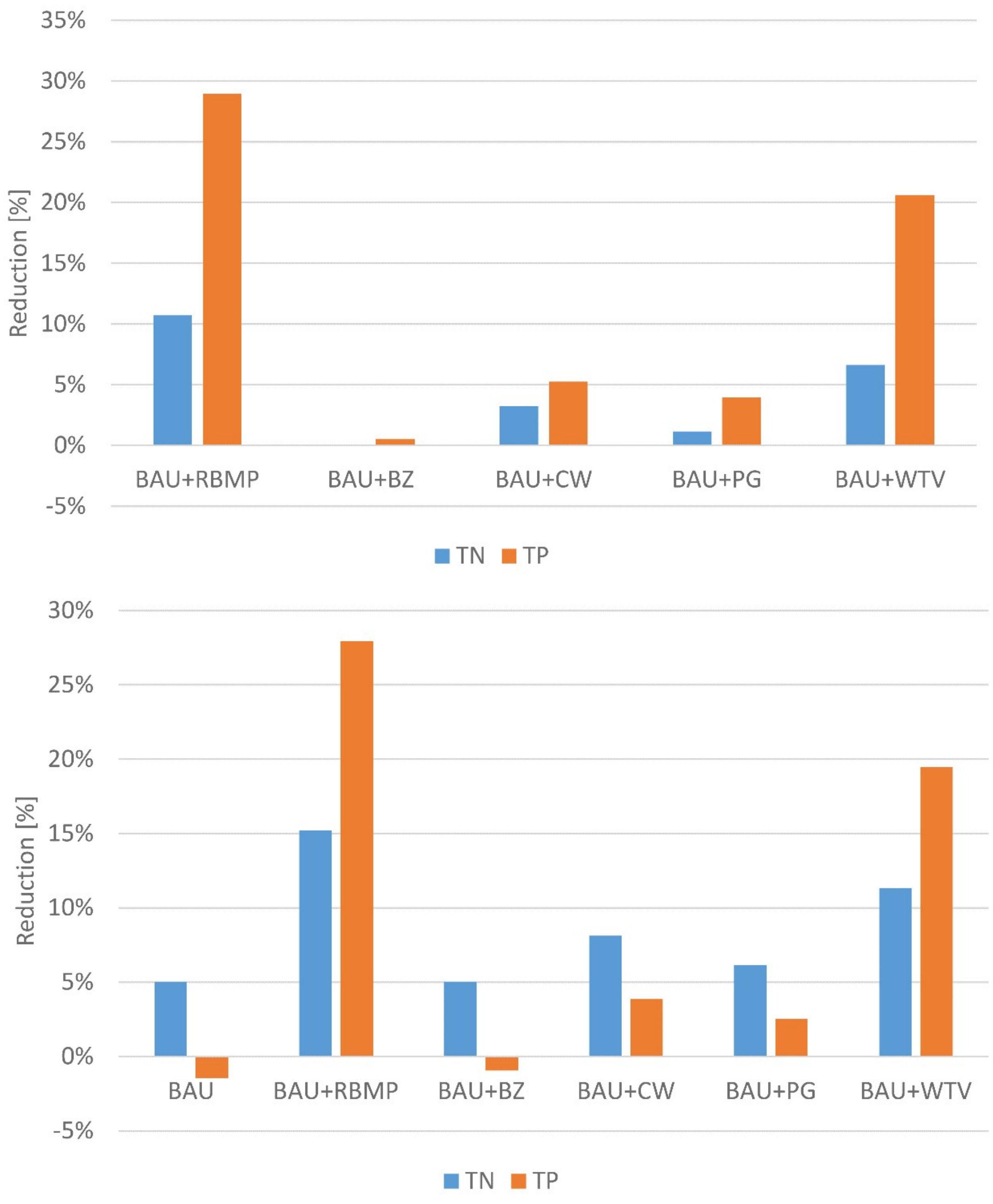

Figure S1 Decomposition of BAU+RBMP scenario for Vantaanjoki catchment. Average reduction of TN and TP loads for different measures (BAU = reference) - upper panel - and average reduction of TN and TP loads for different measures (Baseline = reference) – lower panel. Applying all four measures on top of the BAU scenario leads to a 11% reduction of TN and 29% reduction of TP at the catchment outlet. Decomposition of this scenario into four individual measures shows that the winter-time vegetation is the most efficient for both N and P, whereas buffer zones are the least efficient. According to simulation results the winter-time vegetation helps reduce erosion quite a lot and reduces organic forms of P. BAU – Business-As-Usual; RBMP – River Basin Management Plan; BZ – Buffer Zones; CW – Constructed Wetlands; PG – Perennial Grasses; WTV – Winter-time Vegetation Cover.

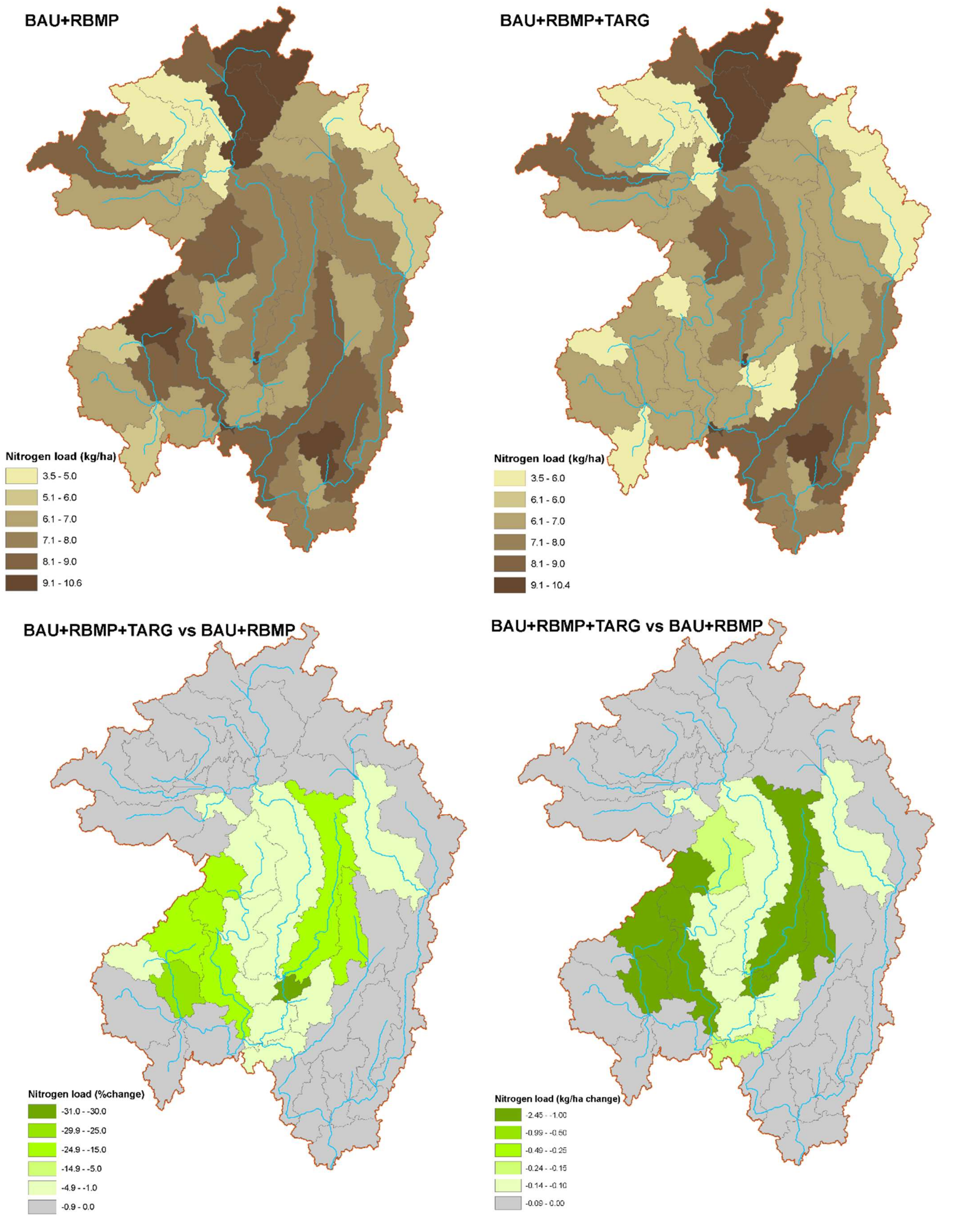

Figure S2 Mean annual **TN** emission at sub-basin level for the **Vantaanjoki** catchment in the BAU+RBMPP and BAU+RBMP+targ scenarios (upper panel). Average decrease in TN emission in BAU+RBMP+targ scenario as compared to BAU+RBMP scenario expressed in percent (left hand site) and in absolute values (right hand site) – lower panel.

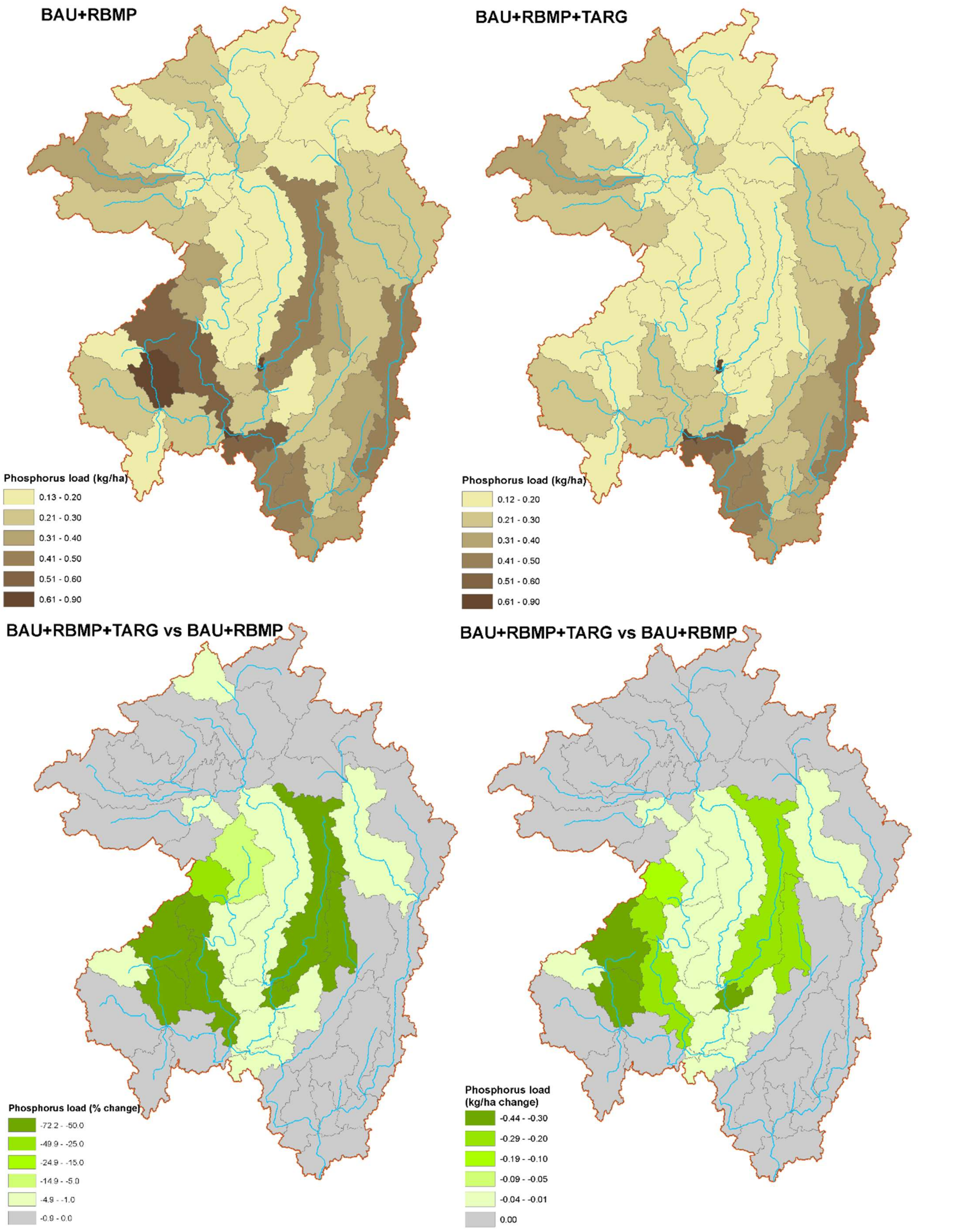

Figure S3 Mean annual **TP** emission at sub-basin level for the **Vantaanjoki** catchment in the BAU+RBMPP and BAU+RBMP+targ scenarios (upper panel). Average decrease in TN emission in BAU+RBMP+targ scenario as compared to BAU+RBMP scenario expressed in percent (left hand site) and in absolute values (right hand site) – lower panel.

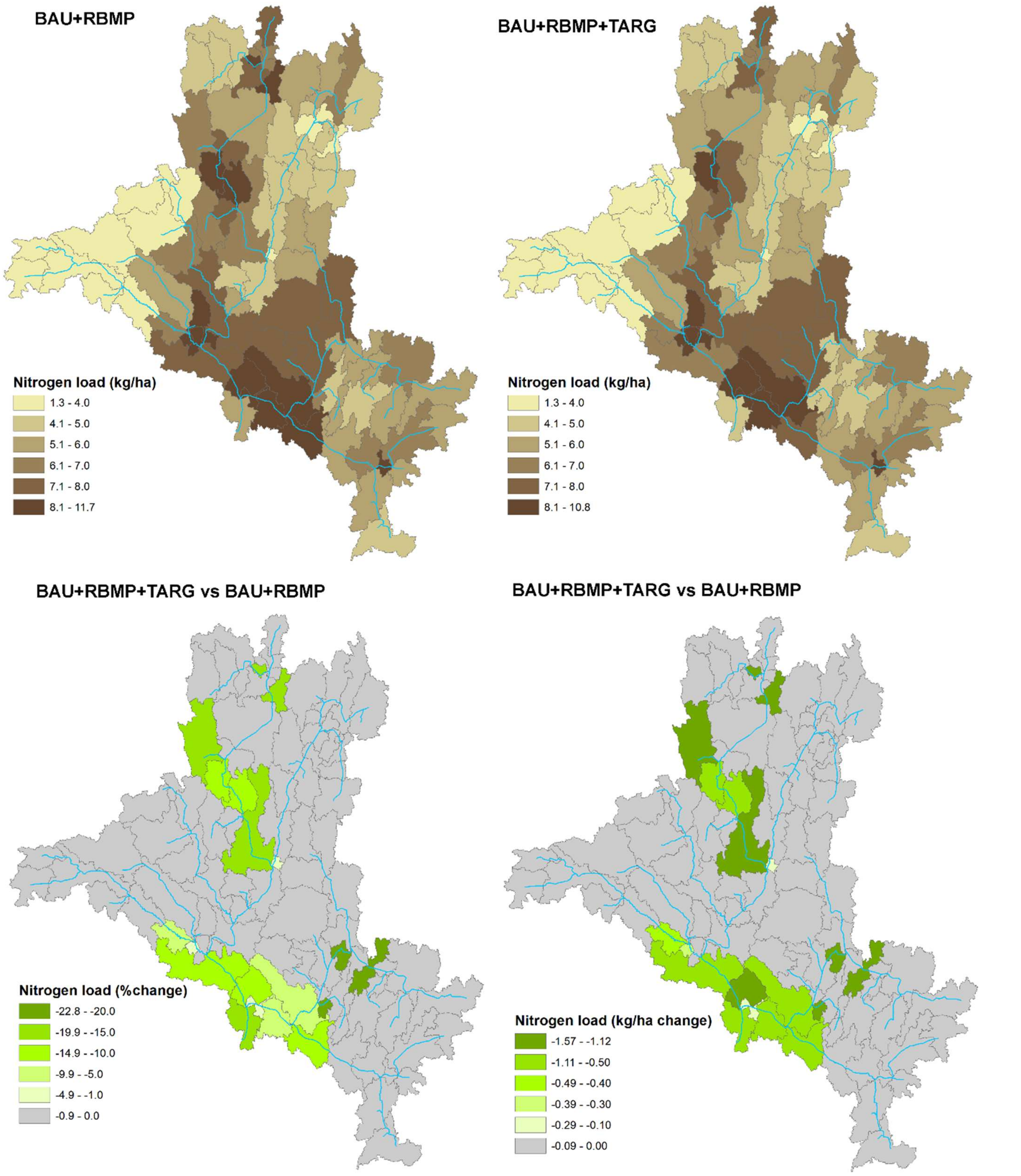

Figure S4 Mean annual **TN** emission at sub-basin level for the **Fyrisån** catchment in the BAU+RBMPP and BAU+RBMP+targ scenarios (upper panel). Average decrease in TN emission in BAU+RBMP+targ scenario as compared to BAU+RBMP scenario expressed in percent (left hand site) and in absolute values (right hand site) – lower panel.

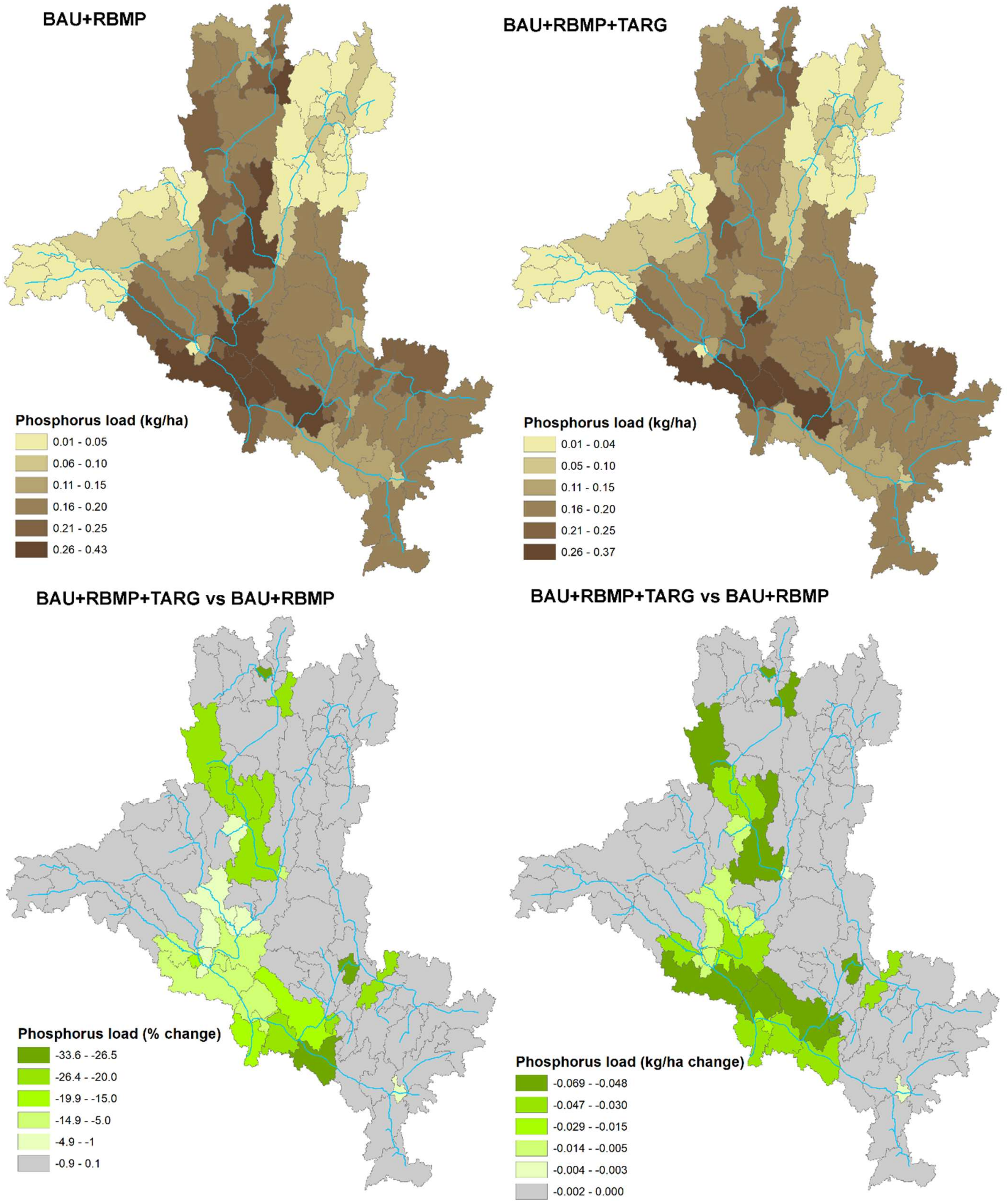

Figure S5 Mean annual **TP** emission at sub-basin level for the **Fyrisån** catchment in the BAU+RBMPP and BAU+RBMP+targ scenarios (upper panel). Average decrease in TN emission in BAU+RBMP+targ scenario as compared to BAU+RBMP scenario expressed in percent (left hand site) and in absolute values (right hand site) – lower panel.

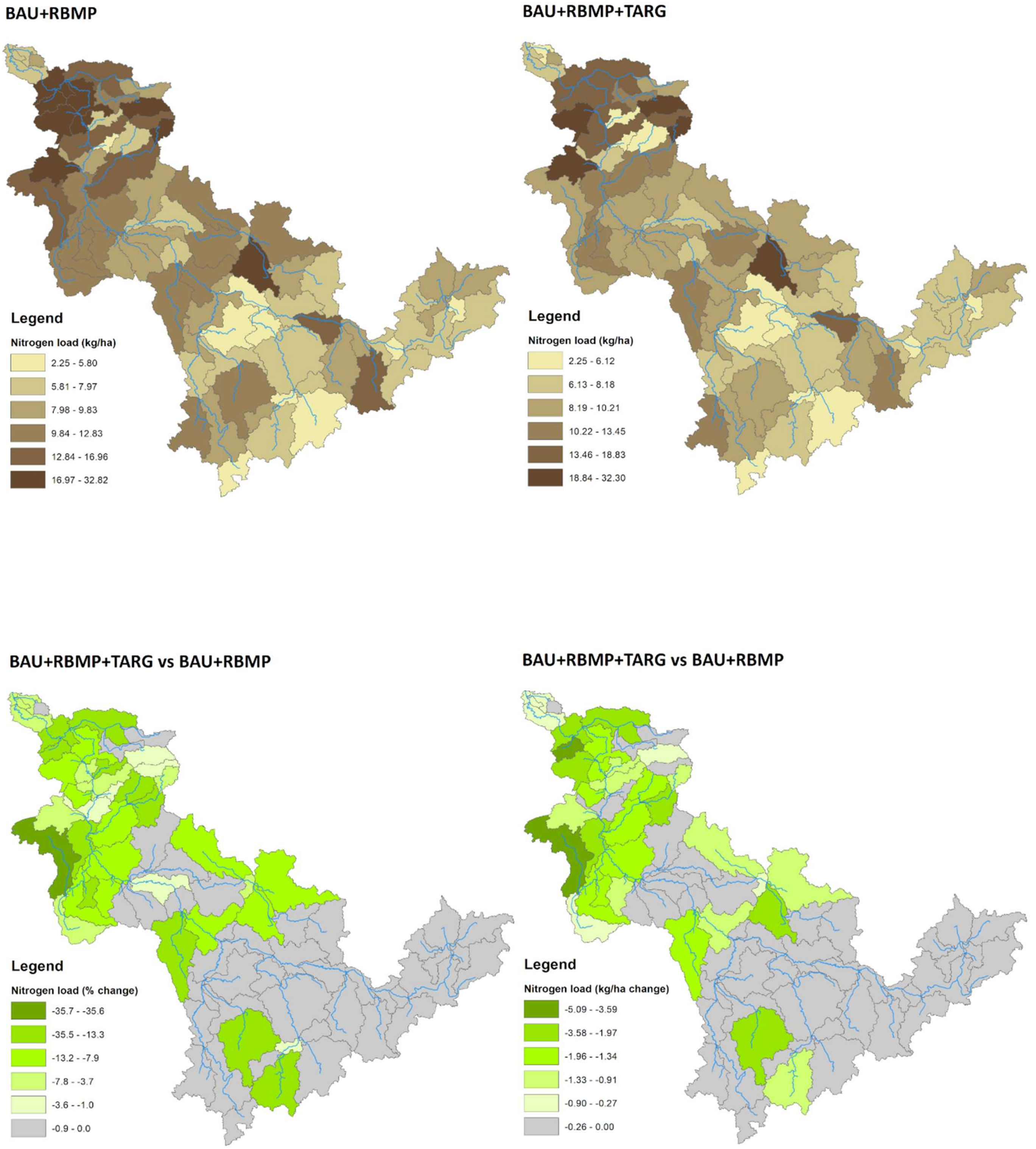

Figure S6 Mean annual **TN** emission at sub-basin level for the **Słupia** catchment in the BAU+RBMPP and BAU+RBMP+targ scenarios (upper panel). Average decrease in TN emission in BAU+RBMP+targ scenario as compared to BAU+RBMP scenario expressed in percent (left hand site) and in absolute values (right hand site) – lower panel.

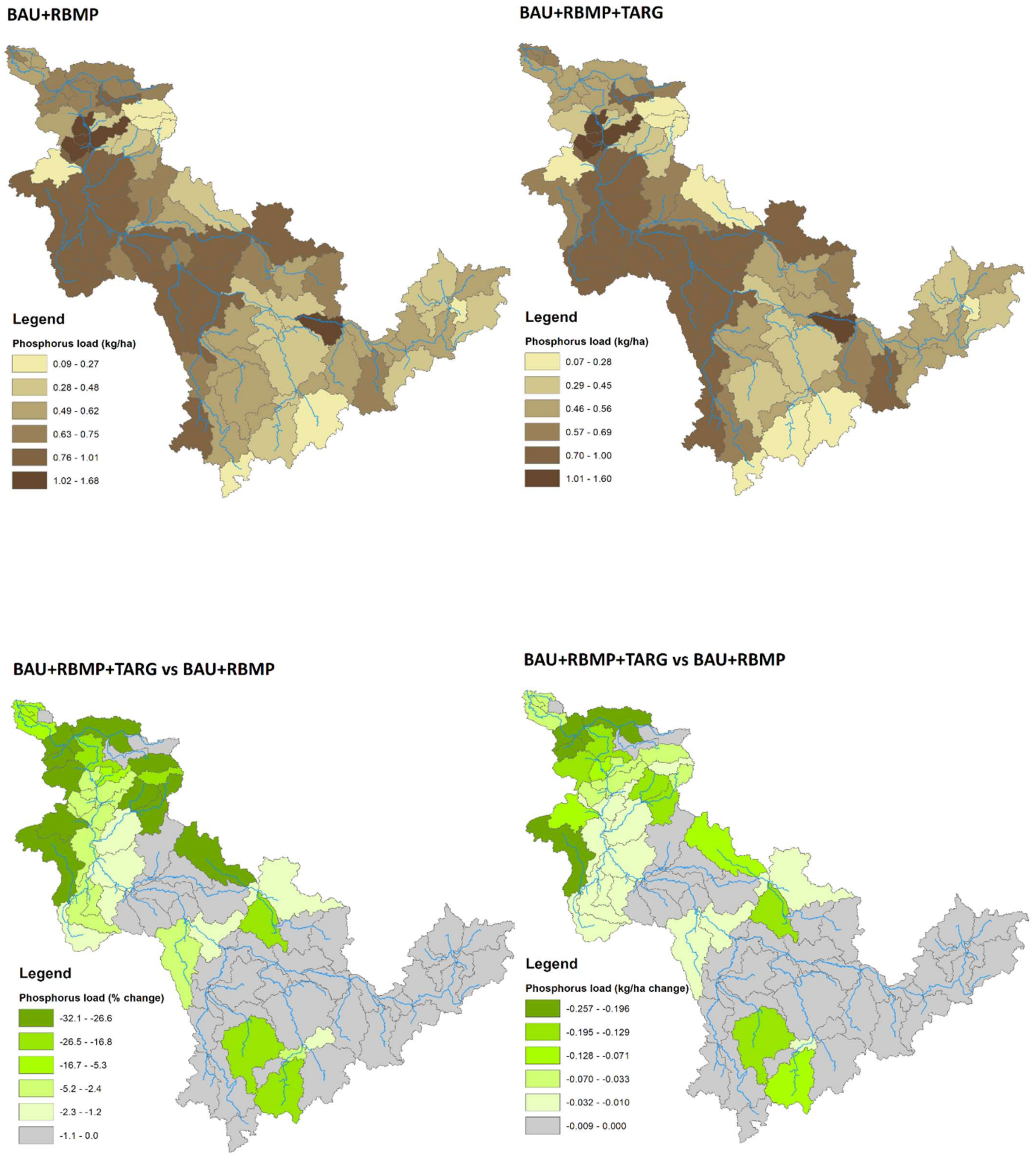

Figure S7 Mean annual **TP** emission at sub-basin level for the **Słupia** catchment in the BAU+RBMPP and BAU+RBMP+targ scenarios (upper panel). Average decrease in TN emission in BAU+RBMP+targ scenario as compared to BAU+RBMP scenario expressed in percent (left hand site) and in absolute values (right hand site) – lower panel.
